# Effectiveness of a Motivational Smoking Reduction Strategy Across Socioeconomic Status and Stress Levels

**DOI:** 10.3389/fpsyg.2022.801028

**Published:** 2022-03-18

**Authors:** Elizabeth C. Voigt, Elizabeth R. Mutter, Gabriele Oettingen

**Affiliations:** ^1^Department of Global Public Health, New York University, New York, NY, United States; ^2^Department of Psychology, New York University, New York, NY, United States

**Keywords:** smoking, socioeconomic status, stress, motivation, mental contrasting with implementation intentions, behavior change, self-regulation, smoking reduction

## Abstract

Smoking consequences are seen disproportionately among low-SES smokers. We examine the self-regulatory strategy of mental contrasting with implementation intentions (MCII) as a smoking reduction tool and whether its effectiveness depends on subjective-SES. This pre-registered online experiment comprised a pre-screening, baseline survey, and follow-up. Participants reported past-week smoking, subjective-SES, perceived stress, and were randomized to an active control (*n* = 161) or MCII condition (*n* = 164). Data were collected via MTurk, during the U.S.’ initial wave of COVID-19. Participants were moderate-to-heavy smokers open to reducing or quitting. The primary outcome was self-reported smoking reduction, computed as the difference between recent smoking at baseline and follow-up. The secondary outcome was cessation, operationalized as self-reported 7-day point-prevalence abstinence at follow-up. Among those low—but not high—in subjective-SES, MCII (vs. control) improved smoking reduction by an average of 1.09 fewer cigarettes smoked per day, though this effect was not conclusive (*p* = 0.11). Similarly, quitting was descriptively more likely for those in the MCII than control condition, but the effect was non-significant (*p* = 0.11). Per an exploratory analysis, we observed that stress significantly moderated the condition effect (*p* = 0.01), such that MCII (vs. control) facilitated reduction among those experiencing high (*p* = 0.03), but not low stress (*p* = 0.15). Consistent with prior findings that MCII works best in vulnerable populations, MCII may be more effective for smoking reduction among high-stress than low-stress individuals. These findings contribute to growing research on income-related health disparities and smoking behavior change tools.

## Introduction

Cigarette smoking is a social justice issue: low socioeconomic-status (SES) individuals bear a disproportionate burden of death and disease (Healton, 2001). Although effective tobacco control policies exist, the equity impact of most is uncertain (Brown et al., 2014), and few specifically target low-SES groups (Hiscock et al., 2012). From a social justice standpoint, research vetting a smoking behavior change strategy should include an examination of whether it is equally, if not more, effective among individuals of low SES. Otherwise, inequitable treatments may further health disparities. Therefore, we explore SES and, on an exploratory basis, perceived stress as potential moderators of a brief behavioral strategy, Mental Contrasting with Implementation Intentions (MCII), that has gathered recent support to reduce smoking.

Low-SES smokers face distinct barriers to quitting and reducing smoking (US Department of Health and Human Services., 2014; Sherman et al., 2016; Centers for Disease Control and Prevention., 2017; Rogers et al., 2019) that may not be addressed in generic treatments. For example, low-SES smokers may experience more stressors (e.g., employment insecurity) and have fewer coping resources (e.g., supportive environments), making quitting or reducing especially difficult (Businelle et al., 2010). There is a need to analyze whether and how existing and emerging tools can help low-SES smokers reduce smoking successfully despite these challenging circumstances.

MCII is a short and practical behavior change strategy that people can self-employ during everyday life. This tool is highly accessible (i.e., brief, little-to-no cost, delivered online) and customizable to personal needs. MCII has been effective across life domains including the health domain (Stadler et al., 2010; Gollwitzer et al., 2018; Valshtein et al., 2020). This thought-based strategy contains two complementary phases—mental contrasting (MC) and implementation intentions (II)—that facilitate binding goal commitments and goal-directed action.

The first step of MC is to name an important, feasible wish and imagine the best outcome of fulfillment. These positive fantasies are juxtaposed with thoughts of one’s inner obstacle standing in the way (Oettingen, 2012). For example, a person may Wish to reduce smoking by half, then identify having more money as the best Outcome. Next, they contemplate their inner Obstacle to cutting back: their tendency to alleviate stress by smoking. Upon discovering one’s inner obstacles via MC (Kappes and Oettingen, 2014), strong associative links form between the outcome, obstacle, and instrumental behavior to overcome the obstacle. These non-conscious links translate into energization (Oettingen et al., 2009) and commitment to wish realization (Oettingen et al., 2001), whereby people more readily perform obstacle-surmounting behaviors (Kappes et al., 2012a,^2013^).

IIs are goal-directed action Plans in the form of an “if…situation, then I will…behavior” statement (Gollwitzer and Sheeran, 2006). The person who identified smoking to cope with stress as their obstacle might form the following II: “If I feel stressed, then I will meditate.” IIs strengthen the associative link between obstacles and instrumental responses, helping when strong impulses arise (Eder, 2011). Thus, MC and II combine into a personalizable tool, tailored to idiosyncratic struggles.

There is limited yet promising research on MCII as a smoking behavior change strategy. When given MC, smokers with high expectations of success took more immediate action to reduce smoking (Oettingen et al., 2010b). When given II, smokers were likelier to quit (Armitage, 2016). And, one study found preliminary evidence that MCII facilitated smoking reduction for highly dependent smokers (Mutter et al., 2020). Despite this promise, MCII’s effectiveness in this domain remains inconclusive.

We are interested in determining whether MCII’s effectiveness as a brief smoking reduction strategy depends on SES. MCII has increased goal striving in different populations (Oettingen and Sevincer, 2018) and has been particularly effective for individuals facing very challenging circumstances. For example, MCII reduced stress in healthcare workers (Gollwitzer et al., 2018), improved homework in ADHD-prone schoolchildren (Gawrilow et al., 2013), and attenuated alcohol consumption in hazardous drinkers (Wittleder et al., 2019). Given their relative lack of resources, low-SES individuals may especially benefit from creative and integrative problem-solving, as well as better time management, all of which are facilitated by mental contrasting (Oettingen et al., 2010a,^2012^, ^2015^; Kirk et al., 2011). Although we might expect a substantial effect of MCII among low-SES smokers based on these findings, limited research has specifically examined MCII’s efficacy with respect to SES. Some evidence suggests that MCII should be at least as effective among low-SES individuals (Gollwitzer et al., 2011; Duckworth et al., 2013; Sheeran et al., 2013); however, no study to date has included a higher-SES comparison group.

To this question, we obtained exploratory evidence suggesting that MCII improves smoking reduction only among *high*-SES individuals in a reanalysis of Mutter et al. (2020) publicly available dataset.^1^ We found that their reported interaction between condition (MCII vs. control) and cigarette dependence was further moderated by subjective-SES (see Supplementary Appendix). Given these conflicting indications, there is a need to examine whether the effect of MCII on smoking reduction is SES-dependent.

In a sample of moderate-to-heavy smokers, we examine the effectiveness of MCII, vs. an active control strategy, as a brief, online tool for smoking reduction, and further test whether its effectiveness depends on subjective-SES. Based on our reanalysis of relevant past data (Mutter et al., 2020), we pre-registered an exploratory hypothesis that MCII would be more effective for high- than low-SES individuals. However, given its exploratory nature and limited research to inform this prediction, we were unsure for whom MCII may work best. We planned to test for similar effects on smoking cessation and explore perceived stress as an additional factor.

Highly stressed individuals may use MCII to address their stress and smoking in tandem, as described earlier and supported by research finding that MCII helps individuals downregulate undesired emotions (Schweiger-Gallo et al., 2018). MCII also helps protect self-appraisals of competence considering setbacks (Kappes et al., 2012b), which could decrease reliance on smoking to manage stressful situations. Thus, MCII should be effective at high levels of perceived stress. For those low in stress, however, who presumably face less dire obstacles to smoking reduction, an active control strategy may be just as effective.

We focus on subjective-SES because it predicts unique variance in self-rated health, above and beyond objective indicators (Cundiff and Matthews, 2017; Zell et al., 2018). Additionally, Mutter et al. (2020) used a subjective measure, so we include it for comparison.

## Materials and Methods

### Participants

Adult participants were recruited using the online platform MTurk (Buhrmester et al., 2011). Eligible respondents were current smokers, reporting an average of 15 + cigarettes per day (CPD) to meet a threshold of moderate smoking (Wilson et al., 1999). Eligible respondents also needed to report an openness to reduce or quit smoking in the next 4 weeks and pass an attention check. Of the 5,685 respondents, 332 met the eligibility criteria and were invited to participate in the full study.^2^ Of these, 325 enrolled and were randomized to condition. Participants were 40.48 years old on average (Winsorized; *SD* = 11.18), majority female (52.9%) and White (76.0%) (for detailed demographics, see Supplementary Table 1).

### Design and Procedure

This study was a pre-registered^3^ online experiment comprising three surveys: pre-screening (T0), baseline (T1), and 4-week follow-up (T2). At the start of T1, after informed consent, participants were randomly allocated via restricted randomization in Qualtrics to either the MCII or control strategy. Subjective-SES was our key moderator of interest. The primary outcome was T1-to-T2 smoking reduction, and the secondary outcome was smoking cessation.

Data were collected from March to August 2020, during the initial U.S. wave of COVID-19. The T0 screener was posted on MTurk as a study called “Your Health and Habits Over Time.” Upon completion, respondents were informed of their (in) eligibility for the full study and were compensated with $0.10 regardless.

**FIGURE 1:**
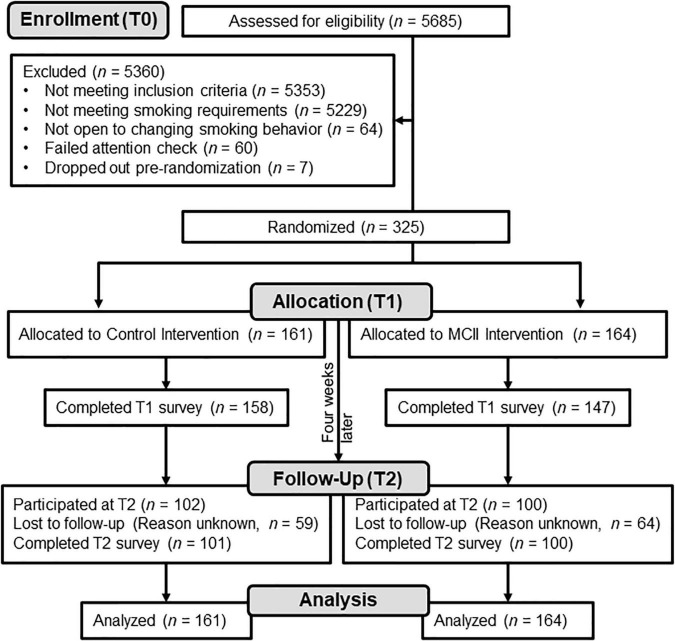
Participant flow diagram. MCII, mental contrasting with implementation intentions.

Those who consented at T1 were enrolled and allocated to an experimental condition or a control condition (control: *n* = 161; MCII: *n* = 164; see Figure 1). Participants were introduced to the “health, wellbeing, and cigarette smoking” study and reported subjective-SES, past-week smoking, and perceived stress. After, participants engaged in their respective strategy. Upon T1 completion, participants were compensated with $1.70 and, 3 days later, sent a reminder of the strategy instructions. Average duration to complete both the T0 and T1 surveys was 18.29 min (Winsorized; *SD* = 10.56).

Participants were invited to complete the T2 survey 4 weeks later.^4^ T2 concluded with a debriefing and $0.50 payment (Winsorized duration, minutes: *M* = 2.48, *SD* = 1.26). Additional methodological details (e.g., auxiliary measures; reminder messages) are reported in Supplementary Appendix, and materials and data are available here: https://osf.io/fzhrj/?view_only=723f8355f4b34c4981fd36285413d338.

### Strategy Condition

Both strategies were self-administered by participants within the online, T1 survey. The instructions they read and responded to at their own pace were adapted from previous research (Marquardt et al., 2017; Wittleder et al., 2019; Mutter et al., 2020) and are described below. Full text of the instructions and example responses for both conditions are available in Supplementary Appendix.

In the MCII condition, participants identified a personally important wish or goal regarding reducing or quitting cigarette smoking in the next 4 weeks. Then, participants identified and imagined the best outcome of attaining their wish and the main inner obstacle “that might stand in the way.” They specified a behavior to overcome this obstacle and created a plan in this format: “If (I encounter my inner Obstacle), then I will (perform the specified behavior to overcome it)!.” Participants were told they learned a strategy to “address wishes and goals,” and reviewed what they had written for each step. To teach them that MCII can be applied to any wishes they might have (Wittleder et al., 2019; Mutter et al., 2020), participants completed another round of MCII, for a shorter-term smoking-related wish.

Control condition participants learned an active strategy that was originally developed from a U.S. government-promoted quit-smoking resource (National Cancer Institute’s Tobacco Control Research Branch) based on motivational interviewing methods. Like MCII, this strategy prompts individuals to contemplate a better future with reduced or absent smoking (Mutter et al., 2020). Specifically, control participants responded to five open-ended questions about their “reasons for reducing or quitting smoking” (e.g., “What do you dislike about smoking that makes you want to quit or reduce?”) Participants reviewed their responses and were informed that they had learned a strategy to identify “reasons for reducing or quitting smoking.”

### Baseline Measures (T1)

Participants reported demographic information including subjective-SES, measured with the MacArthur Scale of Subjective Social Status: “Imagine that the following ladder represents where people stand in the U.S., with those at the top being best-off and those at the bottom being worst-off” (1 = *Least money, education, and respected jobs*, 10 = *Most money, education, and respected jobs*; Adler et al., 2000; Adler and Stewart, 2007). Higher scores indicate greater subjective-SES.

For recent cigarette smoking, we used a modified Timeline Followback procedure, a method to gather retrospective self-reports of substance use (Robinson et al., 2014). Participants reported the number of cigarettes smoked on each day of the past week. The high Cronbach’s alpha for these items, 0.98, indicates that participants smoked a consistent number of cigarettes daily. We computed a measure of recent smoking at baseline (i.e., T1 CPD) by averaging.^5^

We assessed past-month perceived stress to potentially explain a condition-by-SES interaction. Participants completed three items from the Perceived Stress Scale (e.g., “How often have you felt that you were unable to control the important things in your life?”; 0 = *Never*, 4 = *Very Often*; α = 0.70; Cohen et al., 1983), which we averaged for a composite after reverse-scoring one item.

### Outcome Measures (T1 and T2)

At T2, participants were asked whether they had “smoked a cigarette, even just a puff” in the past week. If so, they reported their CPD for the past 7 days. Participants’ T2 CPD score was either 0, if they had not smoked at all, or an average of their past-week smoking (α = 0.99). For smoking reduction, we computed a difference score by subtracting participants’ T2 CPD from their T1 CPD, so that higher scores indicate greater reduction. We defined cessation as self-reported 7-day point-prevalence abstinence (0 assigned if T2 CPD > 0; 1 assigned if T2 CPD = 0; Scheuermann et al., 2017).

### Statistical Analysis

**FIGURE 2:**
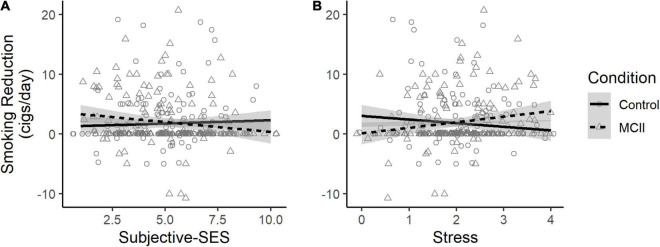
Effect of condition on smoking reduction, depending on **(A)** subjective-SES and **(B)** stress. Jitter was added to better visualize data density. MCII, mental contrasting with implementation intentions; cigs, cigarettes.

Analyses were performed using SPSS (Versions 26–27). Figure 2 was constructed in R. Some auxiliary variables were Winsorized to handle outliers (see Supplementary Appendix). We report 95% confidence intervals (CIs).

For our primary, pre-registered analysis, we regressed smoking reduction on condition (0 = control; 1 = MCII), subjective-SES (mean-centered), and in a subsequent step, their interaction. We planned to follow up with linear contrasts at high (+ 1 *SD*) and low (−1 *SD*) levels of subjective-SES. We pre-registered a secondary, binary logistic regression analysis with the outcome of cessation and the same predictors. Because regression can be sensitive to multivariate outliers, we checked for influential data points using Cook’s distance (0.84 cut-off). All other analyses were exploratory or descriptive. As planned, we did not include any covariates in the regression analyses. Consistent with this approach, we did not find significant differences between the conditions on any baseline smoking-related characteristic (see Table 1).

**TABLE 1:** Descriptive statistics by condition for (a) baseline (Time 1) smoking-related characteristics and (b) key outcomes.

		Control (*n* = 161)	MCII (*n* = 164)	Comparison by condition
**a)**	Smoking frequency,% (*n*)			χ^2^(1) = 1.27
	Some days	12.42 (20)	8.59 (14)	
	Every day	87.58 (141)	92.55 (149)	
	Nicotine replacement frequency,% (*n*)			*r*_*s*_(322) = −0.07
	Not at all	57.76 (93)	63.80 (104)	
	Some days	32.92 (53)	30.06 (49)	
	Every day	9.32 (15)	6.13 (10)	
	Other tobacco products frequency,% (*n*)			*r*_*s*_(322) = −0.02
	Not at all	55.28 (89)	59.51 (97)	
	Some days	32.30 (52)	23.31 (38)	
	Every day	12.42 (20)	17.18 (28)	
	Openness to change,% (*n*)			χ^2^(1) = 0.32
	Open to reducing smoking	64.90 (104)	61.59 (101)	
	Open to quitting smoking	35.40 (57)	38.41 (63)	
	Goal for the next 4 weeks,% (*n*)			χ^2^(1) = 0.04
	Reduce smoking	69.18 (110)	68.10 (111)	
	Quit smoking	30.82 (49)	31.90 (52)	
	Mean start age, in years (*SD*)	17.61 (4.12)	16.94 (3.78)	*t*(320) = 1.52
	Quit attempt during past year,% (*n*)	54.72 (87)	47.24 (77)	χ^2^(1) = 1.80
	Mean quit length, in days (*SD*)	9.49 (10.88)	8.79 (10.25)	*t*(162) = 0.42
	Mean cigarette dependence (*SD*)	19.11 (2.88)	19.62 (2.65)	*t*(316.35) = −1.64
	Mean recent smoking, in CPD (*SD*)	17.83 (7.66)	19.27 (8.72)	*t*(320) = −1.58
	Mean expectations (*SD*)			
	Short-term	3.95 (1.68)	3.77 (1.604)	*t*(321) = 0.99
	Long-term	5.33 (1.36)	5.04 (1.53)	*t*(321) = 1.81
	Mean incentive value (*SD*)			
	Short-term	5.53 (1.48)	5.63 (1.50)	*t*(321) = −0.56
	Long-term	6.04 (1.18)	5.87 (1.48)	*t*(309.60) = 1.12
	Mean WISDM sub-scale scores (*SD*)			
	Social/environmental goads	4.08 (1.87)	4.20 (1.93)	*t*(321) = −0.55
	Cue exposure/associative processes	4.95 (1.42)	5.19 (1.23)	*t*(321) = −1.63
	Mean NDSS priority, in dollars (*SD*)	6.82 (7.22)	6.49 (6.79)	*t*(321) = 0.44
	Experienced SID,% (*n*)	35.22 (56)	36.20 (59)	χ^2^(1) = 0.03
**b)**	Mean smoking reduction, in CPD (*SD*)	1.75 (4.12)	2.02 (4.49)	
	Smoking cessation,% (*n*)	1.90 (3)	4.90 (8)	
	Took an action step,% (*n*)	32.92 (53)	34.15 (56)	

*No comparison in the rightmost column was statistically significant at the α = 0.05 level. Percentages are of those reporting. MCII, mental contrasting with implementation intentions; CPD, average cigarettes per day; WISDM, Brief Wisconsin Inventory of Smoking Dependence Motives; NDSS, Nicotine Dependence Syndrome Scale; SID, smoking induced deprivation; r_s_, Spearman’s rank-order correlation.*

We followed an intent-to-treat approach to missingness (Hollis and Campbell, 1999). Consistent with Mutter et al. (2020), we report findings based on the last-observation-carried-forward approach: participants with missing data at T2 were assigned reduction and cessation scores of 0. Few participants were missing scores on T1 background variables, such as subjective-SES (0.31% missing), perceived stress (0.62%), and CDS (0.92%). We replaced these missing scores with the sample mean.

Because last-observation-carried-forward relies on strict, unverifiable assumptions, and some question the veracity of analyses employing this approach (Little and Yau, 1996; Lachin, 2016), we performed versions of the main analyses with a multiple imputation approach. The results are similar, but slight discrepancies are summarized in Supplementary Appendix.

To determine sample size, we conducted an *a priori* power analysis in G*Power to detect the interaction effect in our primary analysis with 90% power (α = 0.05), with an effect size estimate (f^2^ = 0.037) based on performing our primary analysis in the Mutter et al. (2020) dataset with the present study’s inclusion criteria of average T1 CPD ≥ 15. Accordingly, we recruited participants until at least 290 were randomized to condition at T1.

## Results

Figure 1 depicts participant flow through the study. Table 1 contains descriptive statistics. The experimental groups were similar at baseline. In Supplementary Appendix, we report variations of the analyses adjusting for the few characteristics for which we observed even a trending (*p* < 0.15) difference between-conditions, but the pattern of results is the same as what we report here. Unless stated otherwise, we found no evidence for influential data points.

### Smoking Reduction

The conditions were comparable in T1 subjective-SES (see Supplementary Table 1). As reported in Table 2, there was no main effect of condition or subjective-SES on smoking reduction. The interaction effect neared but did not reach statistical significance (*p* = 0.07; see Figure 2A). Specifically, the planned contrast at low subjective-SES was non-significant but showed that MCII (vs. control) participants reduced smoking by an additional 1.09 CPD, *SE* = 0.68, *t*(321) = 1.62, *p* = 0.11, CI [−0.25, 2.42]. At high subjective-SES, there was also no evidence for a condition effect, *b* = −0.63, *SE* = 0.68, *t*(322) = −0.93, *p* = 0.35, CI [−1.96,0.70].

**TABLE 2:** Multiple regression analyses predicting (a,b) smoking reduction and (c) smoking cessation.

Analysis	Step	Predictor	*b* (*SE*)	*t*	*p*	CI*_*b*_*
**Primary** **(a)**	**Step 1** *F*(2, 322) = 0.60, *p* = 0.55, *R*^2^ = 0.004	Condition	0.23 (0.48)	0.48	0.63	[−0.71, 1.18]
		SES	−0.12 (0.12)	−0.95	0.34	[−0.36, 0.13]
	**Step 2** Δ*F*(1, 321) = 3.23, *p* = 0.07, Δ*R*^2^ = 0.01	Condition	0.23 (0.48)	0.49	0.63	[−0.71, 1.17]
		SES	0.11 (0.17)	0.61	0.54	[−0.24, 0.45]
		Condition by SES	−0.44 (0.24)	−1.80	0.07	[−0.92, 0.04]

**Exploratory** **(b)**	**Step 1** *F*(2, 322) = 0.49, *p* = 0.61, *R*^2^ = 0.003	Condition	0.27 (0.48)	0.56	0.58	[−0.68, 1.21]
		Stress	0.25 (0.30)	0.83	0.41	[−0.34, 0.83]
	**Step 2** Δ*F*(1, 321) = 6.80, *p* = 0.01, Δ*R*^2^ = 0.02	Condition	0.27 (0.47)	0.56	0.58	[−0.67, 1.20]
		Stress	−0.61 (0.44)	−1.38	0.17	[−1.47, 0.26]
		Condition by Stress	1.54 (0.59)	2.61	0.01	[0.38, 2.71]

	**Step**	**Predictor**	***b* (*SE*)**	**χ^2^_*Wald*_**	***p***	**CI (e*^b^*)**

**Secondary** **(c)**	**Step 1** Nagelkerke*R*^2^ = 0.09, x^2^(1) = 7.72, *p* = 0.02	Condition	1.11 (0.69)	2.55	0.11	[0.77, 11.79]
		SES	0.37 (0.16)	5.08	0.02	[1.05, 1.99]
	**Step 2** NagelkerkeΔ*R*^2^ = 0.004	Condition	0.84 (0.79)	1.16	0.28	[0.50, 10.84]
		SES	0.21 (0.30)	0.47	0.49	[0.68, 2.24]
		Condition by SES	0.22 (0.36)	0.38	0.54	[0.62, 2.54]

### Smoking Cessation

Quitting was descriptively likelier for those in the MCII than control condition (see Table 1), but as reported in Table 2, the main effect of condition was non-significant (*p* = 0.11). We also observed a main effect of subjective-SES, such that higher-SES participants were likelier to quit. We found no evidence for a condition-by-SES interaction effect on cessation. One data point exceeded our influence (Cook’s distance) cut-off, so we report an alternative analysis in Supplementary Appendix.

### Interaction With Perceived Stress

We explored whether perceived stress might explain why MCII tended to improve reduction among those with low, but not high, SES. Stress (for descriptives, see Supplementary Table 1) was inversely related to subjective-SES, such that lower-SES individuals were more stressed, *r*(321) = −0.24, *p* < 0.001.

On this basis, we conducted a version of the primary analysis but with stress (mean-centered) as a potential moderator in place of subjective-SES (see Table 2). Stress significantly moderated the condition effect (see Figure 2B), such that MCII (vs. control) facilitated smoking reduction among those high (+ 1 *SD*) but not low (−1 *SD*) in stress (high-stress: *b* = 1.51, *SE* = 0.67, *t*(321) = 2.24, *p* = 0.03, CI [0.19, 2.83]; low-stress: *b* = −0.98, *SE* = 0.67, *t*(321) = −1.46, *p* = 0.15, CI [−2.30, 0.35]).

## Discussion

We sought to examine MCII as a smoking reduction strategy and determine whether its effectiveness, compared to an active control, depends on subjective-SES. Our results suggest this may be the case but are not conclusive. The condition effect at low-SES, though not statistically significant, is consistent with prior findings that MCII helps individuals facing challenging circumstances (e.g., Gawrilow et al., 2013; Gollwitzer et al., 2018; Wittleder et al., 2019; Mutter et al., 2020) and extends prior research on MCII among individuals of low-SES into the domain of smoking reduction.

Our exploratory finding that MCII (vs. the active control) led to greater smoking reduction among those high, but not low, in perceived stress may help explain the pattern we observed regarding SES. Because stress is associated with greater smoking and related vulnerabilities (Pearlin and Schooler, 1978; Cohen and Lichtenstein, 1990; Parrott, 1999; Siahpush et al., 2009), highly stressed individuals—who tended to be of lower-SES in our sample—may stand to benefit the most from this strategy. MCII operates by guiding individuals to discover key obstacles to attaining their wishes and harness energy to overcome them (Oettingen et al., 2009). It may be that participants with high perceived stress had pressing personal obstacles to smoking reduction—perhaps including stress itself—that they could address better with MCII than with the control strategy, thus reaping greater benefits. For low-stress individuals, however, who arguably faced less difficult obstacles, the active control strategy was no less effective than MCII. Future research should confirm whether stress is a reliable moderator of MCII’s effects on smoking reduction and assess relevant mechanisms. As mentioned, these mechanisms may include creative problem-solving, better time management, increased energy, and downregulation of the stress itself.

The small-to-moderate negative association between stress and SES in our sample is unsurprising given the COVID-19 pandemic, which hit low-SES individuals the hardest (Patel et al., 2020). However, in the Mutter et al. (2020) data, the stress-SES correlation was small and non-significant after applying our inclusion criterion (see Supplementary Appendix; see also Almeida et al., 2011, who find a positive stress-SES association). If stress drives the pattern we observed with SES, then this lack of a conclusive stress-SES relationship in the Mutter et al. (2020) data may help explain the different interaction pattern we observed in their data. Future research should investigate perceived stress further: stress may be a better determinant of MCII’s effectiveness in this domain than SES. With SES-related health disparities in mind, however, one might conduct future MCII experimental studies in a population of low-SES individuals undergoing a period of high stress.

Regarding smoking cessation, the descriptively greater prevalence of quitting in the MCII (vs. control) condition is promising but was not conclusive. It is plausible that the study was underpowered to detect effects on cessation, given that only eleven out of 325 participants reported quitting (control: *n* = 3; MCII: *n* = 8). As an important limitation, we relied on a self-report measure of smoking that is psychometrically sound (Robinson et al., 2014) but lacked biochemical verification. Additionally, our 4-week follow-up period makes it difficult to interpret the cessation effects. Future studies may utilize a 6-month follow-up— the standard for cessation studies (Fiore et al., 2008)—allowing participants adequate time to successfully quit, to better test MCII as a cessation tool.

Future research could also investigate MCII in combination with existing interventions, as MCII could easily be layered onto pharmacological or behavioral tools. In fact, combining treatments is considered the gold standard for addressing physical and psychological dependence (Tobacco Use Dependence Guideline Panel., 2008). Nicotine replacement therapy (NRT) is a common pharmacological intervention available through pharmacist-filled prescriptions. Pharmacists play an integral role for patients, and they closely collaborate across teams of healthcare providers (Avalere Health, 2014). When filling prescriptions, pharmacists could alert patients to MCII’s instructions in the form of its colloquial name, WOOP (Wish, Outcome, Obstacle, Plan; see),^6^ and distribute a card with instructions for use with NRT. Similarly, MCII could be administered by clinicians during behavioral counseling sessions or other well-received interventions that increase cessation likelihood (Roberts et al., 2013).

Additionally, it is unclear how the context of the COVID-19 pandemic influenced our results. Participants’ typical smoking patterns may have been disrupted: individuals may have smoked more than usual, under trying circumstances. Or, perhaps some were inclined to reduce smoking due to the increased health risks associated with contracting COVID-19 as a smoker. Regardless, as the pandemic pushes society to become more dependent on and familiar with technology, fully online strategies like MCII merit further study as part of accessible treatment plans.

## Conclusion

Our results were inconclusive regarding the effectiveness of MCII at varying levels of subjective-SES. However, consistent with prior findings that MCII works best in vulnerable populations, our results suggest that MCII may be more effective for smoking reduction among people high in perceived stress than among low-stress individuals. The greater stress experienced by low-SES individuals may have created a vulnerability to smoking that MCII, but not the active control, helped combat against. These findings contribute to the growing body of research on income-related health disparities and smoking behavior change tools.

## Data Availability Statement

The datasets presented in this study can be found in online repositories. The names of the repository/repositories and accession number(s) can be found below: https://osf.io/fzhrj/?view_only=723f8355f4b34c4981fd36285413d338.

## Ethics Statement

The studies involving human participants were reviewed and approved by the University Committee on Activities Involving Human Subjects (UCAIHS), New York University. Written informed consent was not provided because consent for this study was obtained online instead, as was made aware to and approved by the IRB office. The IRB protocol for this study was initiated prior to the Revised Common Rule, so an explicit waiver of written informed consent was not part of the procedure.

## Author Contributions

EV, EM, and GO contributed to the conception and design of the study. EV and EM organized the data, performed the statistical analysis, and wrote sections of the manuscript. EV wrote the first draft of the manuscript. All authors contributed to manuscript revision, read, and approved the submitted version.

## Conflict of Interest

The authors declare that the research was conducted in the absence of any commercial or financial relationships that could be construed as a potential conflict of interest.

## Publisher’s Note

All claims expressed in this article are solely those of the authors and do not necessarily represent those of their affiliated organizations, or those of the publisher, the editors and the reviewers. Any product that may be evaluated in this article, or claim that may be made by its manufacturer, is not guaranteed or endorsed by the publisher.

## Footnotes

1
https://osf.io/spf6a/

2 Of 5,824 participants assessed for eligibility, we removed 139 entries that were not uniquely identified by an MTurk Worker ID, yielding 5,685 unique respondents.

3
https://osf.io/24bfk/?view_only=9c1e696664074d0ebc2c12c673838a4f

4 A greater proportion of control participants (0.98) than MCII participants (0.90) completed the T1 survey, χ^2^(1, N = 325) = 10.17, p = 0.001 (see Figure 1). However, neither participation nor completion at T2 differed by condition [participation: χ^2^(1, N = 325) = 0.20, p = 0.66; completion, of those participating: χ^2^(1, N = 202) = 0.99, p = 0.32]. Missing a T2 recent smoking score was associated with several baseline variables (see Supplementary Table 5) but was not associated with condition, χ^2^(1) = 0.76, p = 0.38.

5 All participants reported at least four of the 7 days; however, we noted three extremely high scores (≥ 120 CPD) indicating a data entry error and dropped them when aggregating those individuals’ responses.

6
woopmylife.org

## References

[B1] Adler N. E. Epel E. S. Castellazzo G. Ickovics J. R. (2000). Relationship of subjective and objective social status with psychological and physiological functioning: preliminary data in healthy, White women. *Health Psychol.* 19:586. 10.1037/0278-6133.19.6.586 11129362

[B2] Adler N. E. Stewart J. (2007). *”The MacArthur Scale of Subjective Social Status.”.* San Francisco: macArthur Research Network on SES & Health.

[B3] Almeida D. M. Piazza J. R. Stawski R. S. Klein L. C. (2011). ““The speedometer of life: stress, health and aging.”,” in *Handbook of the Psychology of Aging*, eds Schaie K. W. Willis S. L. (Amstardam: elsevier), 191–206. 10.1016/B978-0-12-380882-0.00012-7

[B4] Armitage C. J. (2016). Evidence that implementation intentions can overcome the effects of smoking habits. *Health Psychol.* 35 935. 10.1037/hea0000344 27054302

[B5] Avalere Health L. L. C. (2014). *“Exploring Pharmacists’ Role in a Changing Healthcare Environment.”.* Available online at http://assets.fiercemarkets.net/public/Pharmacist%20Report.pdf (accessed January 26, 2022).

[B6] Brown T. Platt S. Amos A. (2014). Equity impact of population-level interventions and policies to reduce smoking in adults: a systematic review. *Drug Alcohol Depend.* 138 7–16. 10.1016/j.drugalcdep.2014.03.001 24674707

[B7] Buhrmester M. Kwang T. Gosling S. D. (2011). Amazon’s Mechanical Turk: a new source of inexpensive, yet high-quality, data? *Perspect. Psychol. Sci.* 6 3–5. 10.1177/1745691610393980 26162106

[B8] Businelle M. S. Kendzor D. E. Reitzel L. R. Costello T. J. Cofta-Woerpel L. Li Y. (2010). Mechanisms linking socioeconomic status to smoking cessation: a structural equation modeling approach. *Health Psychol.* 29:262. 10.1037/a0019285 20496980 PMC2922845

[B9] Centers for Disease Control and Prevention. (2017). *”Current Cigarette Smoking Among Adults in the United States.* Available online at https://www.cdc.gov/tobacco/data_statistics/fact_sheets/adult_data/cig_smoking/index.htm (accessed November 13, 2020).

[B10] Cohen S. Kamarck T. Mermelstein R. (1983). “A global measure of perceived stress.”. *J. of Health Soc. Behav.* 24 385–396. 10.2307/2136404 6668417

[B11] Cohen S. Lichtenstein E. (1990). Perceived stress, quitting smoking, and smoking relapse. *Health Psychol.* 9:466. 10.1037/0278-6133.9.4.466 2373070

[B12] Cundiff J. M. Matthews K. A. (2017). Is subjective social status a unique correlate of physical health? A meta-analysis. *Health Psychol.* 36:1109. 10.1037/hea0000534 28726474 PMC5709157

[B13] Duckworth A. L. Kirby T. A. Gollwitzer A. Oettingen G. (2013). “From fantasy to action: mental contrasting with implementation intentions (MCII) improves academic performance in children.”. *Soc. Psychol. Personal. Sci.* 4 745–753. 10.1177/1948550613476307 25068007 PMC4106484

[B14] Eder A. B. (2011). Control of impulsive emotional behaviour through implementation intentions. *Cogn. Emot.* 25 478–489. 10.1080/02699931.2010.527493 21432688

[B15] Fiore M. C. Jaén C. R. Baker T. B. Bailey W. C. Benowitz N. L. Curry S. J. (2008).

[B16] Gawrilow C. Morgenroth K. Schultz R. Oettingen G. Gollwitzer P. M. (2013). Mental contrasting with implementation intentions enhances self-regulation of goal pursuit in schoolchildren at risk for ADHD. *Motiv. Emot.* 37 134–145. 10.1007/s11031-012-9288-3

[B17] Gollwitzer A. Oettingen G. Kirby T. A. Duckworth A. L. Mayer D. (2011). Mental contrasting facilitates academic performance in school children. *Motiv. Emot.* 35 403–412. 10.1007/s11031-011-9222-0

[B18] Gollwitzer P. M. Mayer D. Frick C. Oettingen G. (2018). Promoting the self-regulation of stress in health care providers: an internet-based intervention. *Front. Psychol.* 9:838. 10.3389/fpsyg.2018.00838 29962979 PMC6013563

[B19] Gollwitzer P. M. Sheeran P. (2006). Implementation intentions and goal achievement: a meta-analysis of effects and processes. *Adv. Exp. Soc. Psychol.* 38 69–119. 10.1016/S0065-2601(06)38002-1

[B20] Healton C. G. (2001). *American Legacy Foundation. Tobacco as a Social Justice Issue. Remarks of Dr. Cheryl Healton.”.* San Francisco: university of California.

[B21] Hiscock R. Bauld L. Amos A. Fidler J. A. Munafò M. (2012). Socioeconomic status and smoking: a review. *Ann. New York Acad. Sci.* 1248 107–123. 10.1111/j.1749-6632.2011.06202.x 22092035

[B22] Hollis S. Campbell F. (1999). What is meant by intention to treat analysis? Survey of published randomised controlled trials. *Bmj* 319 670–674. 10.1136/bmj.319.7211.670 10480822 PMC28218

[B23] Kappes A. Oettingen G. (2014). The emergence of goal pursuit: mental contrasting connects future and reality. *J. Exp. Soc. Psychol.* 54 25–39. 10.1016/j.jesp.2014.03.014

[B24] Kappes A. Oettingen G. Pak H. (2012a). Mental contrasting and the self-regulation of responding to negative feedback. *Personal. Soc. Psychol. Bull.* 38 845–857. 10.1177/0146167212446833 22645162

[B25] Kappes A. Singmann H. Oettingen G. (2012b). Mental contrasting instigates goal pursuit by linking obstacles of reality with instrumental behavior. *J. Exp. Soc. Psychol.* 48 811–818. 10.1016/j.jesp.2012.02.002

[B26] Kappes A. Wendt M. Reinelt T. Oettingen G. (2013). Mental contrasting changes the meaning of reality. *J. Exp. Soc. Psychol.* 49 797–810. 10.1016/j.jesp.2013.03.010

[B27] Kirk D. Oettingen G. Gollwitzer P. M. (2011). Mental contrasting promotes integrative bargaining. *Int. J. Conflic. Manage.* 22 324–341. 10.1108/10444061111171341

[B28] Lachin J. M. (2016). Fallacies of last observation carried forward analyses.”. *Clin. Trials* 13 161–168. 10.1177/1740774515602688 26400875 PMC4785044

[B29] Little R. Yau L. (1996). “Intent-to-treat analysis for longitudinal studies with drop-outs.”. *Biometrics* 52 1324–1333. 10.2307/2532847 8962456

[B30] Marquardt M. K. Oettingen G. Gollwitzer P. M. Sheeran P. Liepert J. (2017). Mental contrasting with implementation intentions (MCII) improves physical activity and weight loss among stroke survivors over one year. *Rehab. Psychol.* 62:580. 10.1037/rep0000104 29265873

[B31] Mutter E. R. Oettingen G. Gollwitzer P. M. (2020). An online randomised controlled trial of mental contrasting with implementation intentions as a smoking behaviour change intervention. *Psychol. Health* 35 318–345. 10.1080/08870446.2019.1634200 31264451

[B32] National Cancer Institute’s Tobacco Control Research Branch (n.d.). *Why You Should Quit: Why Do You Want to Quit?* U.S. Department of Health and Human Services. Available online at https://smokefree.gov/quitting-smoking/reasons-quit/why-do-you-want-quit (accessed July 17, 2017).

[B33] Oettingen G. (2012). Future thought and behaviour change. *Euro. Rev. Soc. Psychol.* 23 1–63. 10.1080/10463283.2011.643698

[B34] Oettingen G. Kappes H. B. Guttenberg K. B. Gollwitzer P. M. (2015). Self-regulation of time management: mental contrasting with implementation intentions. *Euro. J. Soc. Psychol.* 45 218–229. 10.1002/ejsp.2090

[B35] Oettingen G. Marquardt M. K. Gollwitzer P. M. (2012). Mental contrasting turns positive feedback on creative potential into successful performance. *J. Exp. Soc. Psychol.* 48 990–996. 10.1016/j.jesp.2012.03.008

[B36] Oettingen G. Mayer D. Brinkmann B. (2010a). Mental contrasting of future and reality: managing the demands of everyday life in health care professionals. *J. Personnel Psychol.* 9 138–144. 10.1027/1866-5888/a000018

[B37] Oettingen G. Mayer D. Thorpe J. (2010b). Self-regulation of commitment to reduce cigarette consumption: mental contrasting of future with reality. *Psychol. Health* 25 961–977. 10.1080/08870440903079448 20204943

[B38] Oettingen G. Mayer D. Timur Sevincer A. Stephens E. J. Pak H. J. Hagenah M. (2009). “Mental contrasting and goal commitment: the mediating role of energization.”. *Personal. Social Psychol. Bull.* 35 608–622. 10.1177/0146167208330856 19213924

[B39] Oettingen G. Pak H. J. Schnetter K. (2001). Self-regulation of goal-setting: turning free fantasies about the future into binding goals. *J. Personal. Soc. Psychol.* 80:736. 10.1037/0022-3514.80.5.736 11374746

[B40] Oettingen G. Sevincer A. T. (2018). *Fantasy about the future as friend and foe.” The psychology of thinking about the future.* New York: the Guilford Press.

[B41] Parrott A. C. (1999). Does cigarette smoking cause stress? *Am. Psychol.* 54:817. 10.1037/0003-066X.54.10.817 10540594

[B42] Patel J. Nielsen F. B. H. Badiani A. A. Assi S. Unadkat V. A. Patel B. (2020). Poverty, inequality and COVID-19: the forgotten vulnerable. *Public. health* 183:110. 10.1016/j.puhe.2020.05.006 32502699 PMC7221360

[B43] Pearlin L. I. Schooler C. (1978). “The structure of coping.”. *J. Health Soc. Behav.* 19 2–21. 10.2307/2136319 649936

[B44] Roberts N. J. Kerr S. M. Smith S. M. S. (2013). Behavioral interventions associated with smoking cessation in the treatment of tobacco use.”. *Health Serv. Insights* 6:HSI–S11092. 10.4137/HSI.S11092 25114563 PMC4089707

[B45] Robinson S. M. Sobell L. C. Sobell M. B. Leo G. I. (2014). Reliability of the Timeline Followback for cocaine, cannabis, and cigarette use. *Psychol. Addic. Behav.* 28:154. 10.1037/a0030992 23276315

[B46] Rogers E. Vargas E. Rosen M. Barrios-Barrios M. Rana M. Rezkalla J. (2019). “Integrating financial coaching and smoking cessation coaching to reduce health and economic disparities in low-income smokers,” in *Proceedings of the APHA’s 2019 Annual Meeting and Expo (Nov. 2-Nov.6)*. Available online at: https://apha.confex.com/apha/2019/meetingapi.cgi/Paper/439271?filename=2019_Abstract439271.pdf&template=Word (accessed February 27, 2022).

[B47] Scheuermann T. S. Richter K. P. Rigotti N. A. Cummins S. E. Harrington K. F. Sherman S. E. (2017). Accuracy of self-reported smoking abstinence in clinical trials of hospital-initiated smoking interventions.”. *Addiction* 112 2227–2236. 10.1111/add.13913 28834608 PMC5673569

[B48] Schweiger-Gallo I. Bieleke M. Alonso M. A. Gollwitzer P. M. Oettingen G. (2018). Downregulation of anger by mental contrasting with implementation intentions (MCII). *Front. Psychol.* 9 1–10. 10.3389/fpsyg.2018.01838 30337897 PMC6180165

[B49] Sheeran P. Harris P. Vaughan J. Oettingen G. Gollwitzer P. M. (2013). Gone exercising: mental contrasting promotes physical activity among overweight, middle-aged, low-SES fishermen. *Health Psychol.* 32:802. 10.1037/a0029293 22888817

[B50] Sherman S. E. Link A. R. Rogers E. S. Krebs P. Ladapo J. A. Shelley D. R. (2016). Smoking-cessation interventions for urban hospital patients: a randomized comparative effectiveness trial. *Am. J. Prevent. Med.* 51 566–577. 10.1016/j.amepre.2016.06.023 27647057 PMC5089173

[B51] Siahpush M. Yong H. H. Borland R. Reid J. L. Hammond D. (2009). Smokers with financial stress are more likely to want to quit but less likely to try or succeed: findings from the International Tobacco Control (ITC) Four Country Survey. *Addiction* 104 1382–1390. 10.1111/j.1360-0443.2009.02599.x 19438837 PMC2714876

[B52] Stadler G. Oettingen G. Gollwitzer P. M. (2010). Intervention effects of information and self-regulation on eating fruits and vegetables over two years. *Health Psychol.* 29:274. 10.1037/a0018644 20496981

[B53] Tobacco Use Dependence Guideline Panel. (2008). *Treating Tobacco Use and Dependence: 2008 Update.* Rockville (MD): uS Department of Health and Human Services.

[B54] US Department of Health and Human Services. (2014). *The Health Consequences of Smoking—50 Years of Progress: a Report of the Surgeon General.* Atlanta: national Center for Chronic Disease Prevention and Health Promotion.

[B55] Valshtein T. J. Oettingen G. Gollwitzer P. M. (2020). Using mental contrasting with implementation intentions to reduce bedtime procrastination: two randomised trials. *Psychol. Health* 35 275–301. 10.1080/08870446.2019.1652753 31403339

[B56] Wilson D. Parsons J. Wakefield M. (1999). The health-related quality-of-life of never smokers, ex-smokers, and light, moderate, and heavy smokers. *Prevent. Med.* 29 139–144. 10.1006/pmed.1999.0523 10479599

[B57] Wittleder S. Kappes A. Oettingen G. Gollwitzer P. M. Jay M. Morgenstern J. (2019). “Mental contrasting with implementation intentions reduces drinking when drinking is hazardous: an online self-regulation intervention.”. *Health Educ. Behav.* 46 666–676. 10.1177/1090198119826284 30836781

[B58] Zell E. Strickhouser J. E. Krizan Z. (2018). Subjective social status and health: a meta-analysis of community and society ladders. *Health Psychol.* 37:979. 10.1037/hea0000667 30234357

