# Supplementary material for: Effectiveness of a Motivational Smoking Reduction Strategy Across Socioeconomic Status and Stress Levels
Source: Front Psychol. 2022 Mar 18;13:801028. doi: 10.3389/fpsyg.2022.801028 (PMC8973437; doi:10.3389/fpsyg.2022.801028)
Supplement: Supplementary file 1 [file Table_1.DOCX]

Supplementary Material

# Additional Methodological Details

## Recruitment

Based on MTurk’s Worker Requirements, all participants were located in the United States and had a high rate of satisfactory performance (75%) on prior MTurk tasks. Additionally, we temporarily utilized MTurk’s Premium Smoker Qualification: the pre-screening (T0) survey initially required prospective participants to have a premium qualification as ‘smokers.’ Partway through data collection, we removed this qualification requirement and instead relied solely on the pre-screening survey to identify respondents meeting our inclusion criterion of smoking at least 15 CPD. To increase the recruitment rate, we raised the compensation participants received for completing the T1 survey by $0.20, from $1.50 to $1.70. Only the first 100 participants who enrolled in the study and completed the T1 survey were bonused with the original amount of $1.50.

## Survey Timing

All pre-screened and eligible MTurk Workers were given the option to complete the T1 survey at a later date. Those who selected this option were sent an invite through MTurk within a few days of completing the T0 survey to participate in T1 for additional payment at their discretion. Only 19 pre-screened MTurk Workers opted to participate at a later date and 11 of these were randomized to condition. Regarding the follow-up survey, participants were told that within four weeks of completing T1, they would be sent a message via MTurk with a link to complete the final part of the study (T2). For example, a participant who completed T1 on 6/8/2020 was invited to participate in T2 on 7/8/2020. At T2, participants were asked to report their cigarette smoking in the past week and any steps taken toward reducing or quitting smoking.

## Strategy Intervention Condition

Here, we include the full text of the instructions given to participants in the MCII and control conditions, starting with the MCII instructions (wish, outcome, obstacle, plan), as well as examples of qualitative responses. Based on content analysis of responses from previous research, small modifications were made to the wording and bolding of the MCII intervention instructions used by Mutter et al. (2020) to support participant engagement and identification of a truly inner obstacle.

Wish instructions:

Each day as we get up in the morning, go about our business, and go to sleep at night, we think about different wishes in our lives. Think about the next 4 weeks. Regarding reducing or quitting cigarette smoking, what is your **most important wish or goal** you would like to fulfill in these next 4 weeks? Please pick a wish related to reducing or quitting cigarette smoking that is **challenging for you** but that you are fairly **confident** you can fulfill within the next 4 weeks. *Please write it down in a few key words.*

Wish checks: Three questionnaire items were included to check that participants followed these directions to choose an important, feasible wish. They answered the following questions on a seven-point scale (1 = *Not at all*, 7 = *Extremely*):

“How **likely** do you think it is that you will realize your wish or goal over the next 4 weeks?”

“How **important** is it for you to realize your wish or goal over the next 4 weeks?”

“How **disappointed** would you feel if you did not realize your wish or goal over the next 4 weeks?”

Outcome instructions:

What would be the **one best outcome** of realizing your wish or goal related to reducing or quitting cigarette smoking? What would be the most wonderful thing about it? How would you feel?

Instructions after naming the outcome:

Please take a minute or so to think about the best positive outcome you just named, ("${outcome1/ChoiceTextEntryValue/1}"), **in vivid detail**. Letting your mind wander freely, imagine this outcome and **write about all the thoughts and images that come to your mind**. Please take as much time as you need, give your thoughts and images free reign, and write them down:

Obstacle instructions:

Sometimes things do not work out as we would wish. What is it **in you** that might stand in the way of you realizing your wish or goal in the next 4 weeks? What is **your main inner obstacle** (something that is in YOU, in YOUR behavior, that may prevent your wish or goal from becoming reality)?

Instructions after naming the obstacle:

Now, please take a minute or so to think about your inner obstacle you just named, ("${obstacle1/ChoiceTextEntryValue/1}"), in vivid detail. Letting your mind wander freely, imagine your inner obstacle occurring and write about all the thoughts and images that come to your mind. Please take as much time as you need, give your thoughts and images free reign, and write them down:

Plan instructions:

Next, you will form an **if-then plan** to help you in realizing your wish or goal. Recall the inner obstacle you named earlier: "${obstacle1/ChoiceGroup/AllChoicesTextEntry}"

What could YOU do to **overcome** your inner obstacle? What would be an effective action you could take or an effective thought you could think to surmount your inner obstacle? Name one effective action or thought (i.e., "Behavior") below.

Instructions after naming the behavior for the plan:

Now, please form an if-then plan, using the inner obstacle and behavior you've already named, according to the following format: "**If** (I encounter my **inner Obstacle**), **then I will** (perform the **Behavior** to overcome it)!"

Instructions after forming the plan:

**Say this if-then plan slowly to yourself, and imagine acting out the plan.** "If ${plan1/ChoiceTextEntryValue/1}, then I will ${plan1/ChoiceTextEntryValue/2}!"

Explanation of the MCII strategy (Note: MCII is referred to as WOOP in public-facing materials, e.g., www.woopmylife.org):

You just learned the *WOOP* technique used to address wishes and goals people may have.

The key elements follow the acronym **WOOP**:

First, you named an important **W**ISH or goal that is challenging but you are confident you will be able to realize in the next 4 weeks.

Then, you thought about the very best **O**UTCOME of realizing your wish or goal.

Next, you thought about your main inner **O**BSTACLE that stands in the way of realizing your wish or goal.

Finally, you created an if-then **P**LAN to overcome the inner obstacle.

…

Great! Now you have practiced the strategy called WOOP for helping you reach any wishes or goals you might have, including reducing or quitting smoking. These wishes may be big or small, trivial or deep, short-term or long-term — as long as they are important to YOU. You can practice the WOOP technique at any time throughout the day - while you're waiting for the bus, at breakfast, or before you go to sleep.

Instructions leading into the 24-hour wish, after which participants engage in the same steps of MCII for this new, shorter-term wish, guided by the same instructions as for the 4-week wish above, save for the time frame:

You can use WOOP for any wishes or goals you might have. Let's practice applying the technique with a **24-hour wish**.

MCII — Example qualitative responses to the first, 4-week round of MCII and the second round of MCII, for a wish in the next 24 hours (Note: These two sets of responses were taken from two different participants):

**4-week Wish**: I wish I could smoke one cigarette per hour.I have been able to do this before and it would cut me back to smoking half of what I do now.

**4-week Best Outcome**: I would have more self esteem due to reaching my goal.

***Outcome Imagery***: I would have more energy and feel good about myself. It would save me money also. Might be able to sleep better. Sometimes I wake up coughing because of my smoking.

**4-week Obstacle**: Boredom

***Obstacle Imagery***: I smoke alot out of boredom. I really need to find more things to do . Perhaps take up a hobby or clean around the house more. I work outside in my garden in the summer and swim in our pool so I smoke less in the summer.

**4-week Plan**: If I become bored, then I will get up and find something to do.!

**24-hour Wish**: wait 20 minutes after waking up to smoke

**24-hour Best Outcome**: slow my smoking down, and feeling better

***Outcome Imagery***: If I waited 20-30 min every morning before having a cigarette, then I for one would get better sleep according to my doctor. That, will improve my daily functioning and memory because I won't be as tired for lack of sleep.

**24-hour Obstacle**: not believing in myself

***Obstacle Imagery***: I tend to have goals, but I end up letting my negative thoughts take over and tell myself I can't do something, especially if I have tried and failed before.

**24-hour Plan**: If I start to not believe in myself, then I will try to stay positive and have faith!

Control condition instructions: The following are the five questions that were presented, on separate survey pages, to the control condition participants.

1) What do you **dislike** about smoking that makes you want to quit or reduce?

2) What do you **miss out** on when you smoke that makes you want to quit or reduce?

3) How is smoking affecting your **health** in a way that makes you want to quit or reduce?

4) If you don’t quit or reduce smoking, what will happen to **you and your family**?

5) How will your life get **better** when you reduce or quit smoking?

Explanation of the control strategy:

Great! You have just learned a strategy called *Reasons for Quitting* to identify your reasons for reducing or quitting smoking. Now that you know your reasons, you can remind yourself of them every day - while you're waiting for the bus, at breakfast, or before you go to sleep.

Note that these questions are all geared towards reducing or quitting smoking, just like the MCII instructions. Due to an experimenter error, these questions were worded slightly differently in the version of the survey sent to participants who passed the pre-screening but wanted to take the T1 survey later, at a separate time. For example, the first question simply read, “What do you **dislike** about smoking?” Because the primary question content was still present, and because this variation of the survey was only taken by four of the 161 control participants (2.48%), we are unconcerned about it influencing our results.

Control — Example qualitative response:

1) I dislike the social disdain I sense from others because I smoke. I dislike the cost and smell. I dislike the time that it takes away from other activities (i.e., by going outside to smoke instead of doing something else).

2) I am not "allowed" to smoke around my grandchildren, so sometimes I don't get to spend as much time with them as I would like.

3) I am heavy and also have high blood pressure. I also have a history of cancer. I am high risk for many illnesses include heart problems, heart attack, stroke, cancer, etc. I want to live a long and healthy life.

4) I will likely die early, which would have a devastating effect on my husband and children.

5) I won't have to worry about social stigma. I will be able to spend more time with my grandchildren. I will have more spending money. I won't have to deal with the smell. I would be happier with myself. I would be healthier.

## Strategy Reminder

Upon completion of the T1 survey, participants were individually contacted three days later through MTurk’s email system. The message included a bonus and a reminder message of the strategy (WOOP or Reasons for Quitting/Reducing) they had learned to help in realizing their goal of reducing or quitting smoking. Participants were then prompted to use their respective strategy every day. Lastly, we told participants they will be invited to participate in the final part of the study within three and a half weeks. Due to an experimenter error, not all bonuses and reminder emails were sent within the same time frame. While most participants received these materials 3 days after completing T1, some participants received bonuses and strategy reminders up to 7 days after completing T1. Because all participants eventually received the same materials and because the difference was only a few days, we are unconcerned about this impacting our results.

MCII Strategy Reminder:

Hello! Thank you for participating in Part 1 and 2 of the HIT “Your Health and Habits Over Time”. This is your 3-day reminder of the strategy you learned to help you in realizing your goal of reducing or quitting your cigarette smoking. If you recall, you learned a strategy called WOOP, which entailed the following steps: WISH: What is your most important wish or goal that is challenging but you are confident you will be able to realize? Best OUTCOME: What would be the one very best outcome of realizing your wish or goal? Main inner OBSTACLE: What is it in you that might stand in the way of realizing your wish or goal? If-then PLAN: "If (I encounter my inner Obstacle), then I will (perform the Behavior to overcome it)!" You can use WOOP for any wish or goal you might have every day, including wishes and goals regarding reducing or quitting smoking - while you're waiting for the bus, at breakfast, or before you go to sleep. We will be in touch in about 3-1/2 weeks (25 days) with an invitation to participate in the final part of this study, Part 3. Thank you!

Control Strategy Reminder:

Hello! Thank you for participating in Part 1 and 2 of the HIT “Your Health and Habits Over Time”. This is your 3-day reminder of the strategy you learned to help you in realizing your goal of reducing or quitting your cigarette smoking. If you recall, you learned a strategy called Reasons for Quitting, in which you considered and responded to the following questions: What do you dislike about smoking? What do you miss out on when you smoke? How is smoking affecting your health? If you don’t quit or reduce smoking, what will happen to you and your family? How will your life get better when you reduce or quit smoking? You can remind yourself of your reasons for reducing or quitting smoking every day - while you're waiting for the bus, at breakfast, or before you go to sleep. We will be in touch in about 3-1/2 weeks (25 days) with an invitation to participate in the final part of this study, Part 3. Thank you!

## Pre-screening (T0) Eligibility Measures

During the pre-screening survey, participants were first asked about their smoking status: ‘In your entire life, have you ever smoked a total of 100 cigarettes?’ (0 = *No*, 1 = *Yes*; (Baggett et al., 2013). A participant who responded ‘Yes’ was considered an ‘ever-smoker.’ Participants also reported how often they “smoke cigarettes,” use “other tobacco products,” and use “nicotine replacements” (0 = *Not at all*, 1 = *Some days*, or 2 = *Every day*). Those who reported currently smoking cigarettes ‘Every day’ or ‘Some days,’ were considered a ‘current-smoker.’ Only participants who reported being both an ever-smoker and current-smoker were eligible for the T1 survey. The frequency of using other tobacco products and nicotine replacements was collected only to characterize the sample.

To assess whether participants met our eligibility criteria of smoking an average of at least 15 cigarettes per day, participants were next asked, ‘In the past 7 days have you smoked a cigarette, even just a puff?’ (Rogers et al., 2019). Then, they reported, ‘On average, how many cigarettes do you smoke per day? (1 pack = 20 cigarettes),’ by typing a number into a blank text box.

Next, we asked participants if they were ‘open to changing [their] cigarette smoking in the next 4 weeks’ (1 = *Yes, I am open to* ***reducing*** *how many cigarettes I smoke*, 2 = *Yes, I am open to* ***quitting*** *smoking cigarettes*, 3 = *No, I am not open to changing*, 4 = *Other - please specify*). To be eligible, participants needed to indicate their openness to reducing or quitting smoking by choosing option 1 or 2.

As a data quality check, we also included an attention check item: ‘This question assesses whether you are paying attention. Please select the answer option between five and seven’ (1 = *I am a human*, 4 = *Neutral*, 7 = *I am a robot*). To be eligible, participants had to select ‘6.’

## Demographic Measures (T1)

All demographic information was collected at the start of the T1 survey, although we pre-registered to include these measures at T0. Our initial plan was to prevent missing demographic data because subjective-SES was a key independent variable for our analyses. However, we later prioritized keeping the T0 pre-screener as brief as possible which resulted in this switch. Evidently, collecting demographic information at the start of T1 still circumvented participant fatigue as only 1 participant was missing data for the subjective-SES measure.

At T1, participants reported their gender (0 = *Male*, 1 = *Female*, 2 = *Other / non-binary - specify if desired*), age (numerical text entry), and racial and ethnic group identification (instructed to select all that apply: *African-American / Black / African descent*, *American Indian / Native American / Alaskan Native*, *Arab-American / Middle Eastern descent / North African descent*, *Asian-American / Asian descent / Pacific Islander*, *European-American / White / Caucasian / European descent*, *Hispanic-American / Hispanic / Latino(a)(x)*, *Other*, *Unknown*).

We also asked participants about objective measures of socioeconomic status including their highest level of education (1 = *Less than high school*, 2 = *Some high school*, 3 = *High school diploma or GED*, 4 = *Some college*, 5 = *College degree*, 6 = *Associate’s degree*, 7 = *Some graduate or professional training*, 8 = *Graduate or professional degree*), their annual income (1 = *No Income*, 2 = *< $5,000 per year*, 3 = *$5,000 to $11,999*, 4 = *$12,000 to $19,999*, 5 = *$20,000 to $39,999*, 6 = *$40,000 to $59,999*, 7 = *$60,000 to $79,999*, 8 = *$80,000 to $99,999*, 9 = *$100,000 or greater*), and their employment status (instructed to select all that apply from the following: *Working full-time, >35 hours/week*; *Working part-time*; *Freelance/Self-employed*; *Unemployed, seeking work*; *Unemployed, not seeking work*; *Homemaker*; *Disabled*; *Retired*; *Student, full-time*; *Student, part-time*). Because these data were collected during the coronavirus pandemic, we asked participants to check a box if they were ‘Currently working from home’ and to check another box if their ‘Employment status is uncertain.’

## Mental Health Measures (T1)

In addition to the general perceived-stress measure described in the main text, we also included four items to assess participants’ stress specifically in response to the coronavirus pandemic, measured on the same scale: ‘**Due to the present situation,** how often in the last 2 weeks have you felt that…’ 1) ‘You were unable to control the important things **in your life**?’ 2) ‘You were unable to control the important things **in the lives of your loved ones**?’ 3) ‘You were unable to control the important things **in society?**’ and 4) ‘You were **nervous and stressed**?’. These items were also averaged to make a composite (ɑ = .79).

To assess participants’ frequency of experiencing symptoms of anxiety and depression in the last two weeks, we included the four-item Patient Health Questionnaire (PHQ-4; 0 = *Not at All*, 1 = *Several Days*, 2 = *More Than Half the Days*, 3 = *Nearly Every Day*; e.g., ‘Feeling nervous, anxious, or on edge;’(Kroenke et al., 2009). As recommended by Kroenke et al. (2009), we created a composite PHQ-4 score by summing the items (α = .89).

## Auxiliary Smoking-Related Characteristics (T1)

Participants were asked to rate the following indicators of expectations and incentive value, respectively, on a seven-point scale (1 = *Not at all*, 7 = *Very*): ‘How likely do you think it is that you will reduce or quit your cigarette smoking?’ and ‘How important is it to you to reduce or quit your cigarette smoking?’ Both items were presented regarding the short-term (‘In the NEXT 4 WEEKS…’) and the long-term (‘In the LONG-TERM...’), for a total of four items.

To understand participants’ motives for smoking, we included six items from the Wisconsin Inventory of Smoking Dependence Motives (WISDM-68; Piper et al., 2004) These items came from the social/environmental goads subscale (e.g., ‘Most of the people I spend time with are smokers’) and the cue exposure/associative processes subscale (e.g., ‘My life is full of reminders to smoke’). Participants rated their level of agreement with each statement on a seven-point scale (1 = *Not true of me at all*, 7 = *Extremely true of me*). We created a composite score for each subscale by averaging, such that higher scores indicate greater social/environmental goads (α = .94) and stronger cue exposure/associative processes (α = .71), respectively.

Participants were also asked to indicate how much money (in U.S. dollars) they would be willing to pay the next morning for just one cigarette after not being able to get a hold of any. This item was originally developed for the Nicotine Dependence Syndrome Scale (NDSS; Shiffman et al., 2004) but was not included in either the final five-factor or one-factor solutions (see Shiffman et al., 2004, Table 6). We included this item for exploratory purposes because it places a monetary value on participants’ cigarette dependence and thus relates to our interest in smoking behavior change among individuals of varying SES.

To measure cigarette dependence as in Mutter et al. (2020), we included the five-item version of the Cigarette Dependence Scale (CDS-5; Etter et al., 2003). The items asked participants to rate their ‘addiction to cigarettes on a scale of 0-100’ (0 = *I am NOT addicted to cigarettes at all*, 100 = *I am extremely addicted to cigarettes*), how many cigarettes they smoke per day on average, how soon after waking up they smoke their first cigarette (in minutes), how difficult quitting smoking would be (1 = *Very easy*, 2 = *Fairly easy*, 3 = *Fairly difficult*, 4 = *Very difficult*, 5 = *Impossible*), and whether they ‘feel an irresistible urge to smoke’ after ‘a few hours without smoking’ (1 = *Totally disagree*, 2 = *Somewhat disagree*, 3 = *Neither agree nor disagree*, 4 = *Somewhat agree*, 5 = *Fully agree*). Participants who took the T0 pre-screener and the T1 survey in the same sitting had already reported their average CPD, so this original report of CPD in the screener was used in place of the second CDS item, which was omitted for this version of the T1 survey. For participants who took the T1 survey in a separate sitting, the second CDS item was included and used to compute the CDS composite scores.

Responses not already on a five-point scale were recoded to range from 1 to 5, per the recommendations of Etter et al. (2003). Then, item scores were summed such that possible CDS scores range from 5 to 25 (α = .57). Although the scale reliability appears low, the range of CDS scores was effectively truncated by our pre-screening out light smokers, thus dampening inter-item correlations. For descriptive purposes, we defined high cigarette dependence as a composite CDS score of 15 or greater, because this represents the composite scale midpoint.

To measure tobacco-related financial distress, the T1 survey assessed smoking induced deprivation (SID) with a single questionnaire item: ‘In the last 30 days, has there been a time when the money you spent on cigarettes resulted in not having enough money for any of these items: housing, food, household utilities, health care, transportation, and necessary clothing?’ (*Yes* or *No*; Siahpush et al., 2007).

Additional variables characterizing participants’ smoking history and habits were measured for descriptive purposes. We asked participants to report the age they began smoking, if they typically purchase cigarettes by the pack or by the individual cigarette, and how much (U.S. dollars) they ‘typically spend’ on either a pack of cigarettes or a single cigarette, depending on their response to the prior question. We also inquired about any quit attempts made in the past year (whether they have ‘gone one day (or longer) without smoking cigarettes because you were trying to quit’ and if so, for how long, in days).

Finally, we reminded participants that they were invited ‘to take part in this study because [they] expressed an interest in changing [their] cigarette smoking behavior,’ and asked ‘Which statement most applies to [their] goal for the next 4 weeks?’ (1 = *I want to* ***reduce*** *how many cigarettes I smoke*, 2 = *I want to* ***quit*** *smoking cigarettes*).

## Steps Taken (T2)

To explore strategic actions taken by participants, we asked whether they had “taken any steps to reduce or quit cigarette smoking” since they “first enrolled in this study” (0 = *No*, 1 = *Yes*). If “Yes,” participants selected the specific step(s) they had taken from a list of thirteen actions (e.g., “thrown away smoking materials”; for the full list, see Table S3).

## Strategy Recall and Use (T2)

As a check, we also probed recall and use of the intervention strategy. The item regarding strategy use was, “How often have you applied this strategy to your own life since you learned it?” (0 = ** I don’t remember learning a strategy **, 1 = *Never*, 4 = *About half the days*, 7 = *Every day*). The item regarding strategy recall was, “Which strategy did you learn?” (options: *Reasons for Quitting/Reducing*, *WOOP*, *Text 2 Quit*, or *I don’t remember learning a strategy*). For analysis, we created a separate variable capturing whether the participant remembered learning a strategy (1 = gave a non-zero response to strategy use question) or not (0 = responded zero to strategy use question). Then, not remembering values (0’s) were considered missing for the strategy use variable, so that these scores ranged from 1 to 7.

## Winsorizing: Details and Exceptions

For numerical variables whose measurement was not bounded by a set scale, we defined outliers as values exceeding 1.5 IQRs above the 3rd quartile or below the 1st quartile (Hoaglin et al., 2000). We then Winsorized these outliers (i.e., replaced them with the closest non-outlying value), with the following exceptions: We did not Winsorize the variable cigarettes per day (CPD) because our main outcome variables (smoking reduction and smoking cessation) were reliant on reports of the number of cigarettes per day. In particular, reduction scores were dependent on a difference between CPD at T1 and CPD at T2, so a person’s exact reports—so long as they could reasonably indicate a valid report—were important to retain, even if they were atypically high. Additional exceptions were made to exclude the variable “number of days elapsed from T1 to T2” from Winsorization and include the variable “daily spending” in Winsorization, even though its values were dependent on CPD.

# Exploratory Reanalysis of Mutter et al. (2020) Data

## Descriptive Statistics

As reported in the original article (Mutter et al., 2020), average cigarette dependence prior to the intervention was above the scale midpoint of 15 for both conditions, (control: *M* = 16.43, *SD* = 4.71; MCII: *M* = 16.95, *SD* = 4.09; see Mutter et al. (2020) Table 1, p. 331). Average subjective-SES was close to five on a 1-10 scale for both conditions (control: *M* = 4.76, *SD* = 1.70; MCII: *M* = 4.71, *SD* = 1.67; see Mutter et al. (2020) Table S1). There was no evidence that the two intervention groups differed significantly in baseline (T1) cigarette dependence or subjective-SES. Regarding smoking reduction from T1 to T2, participants in both conditions reduced their smoking by an average of more than one cigarette per day (control: *M* = 1.45 cigarettes/day, *SD* = 3.75; MCII: *M* = 1.89 cigarettes/day, *SD* = 4.42; see Mutter et al. (2020) Table 2, p. 332).

## Results: Three-Way Interaction Involving Subjective-SES

We analyzed data using a linear regression approach in IBM SPSS Statistics software (version 26). To test whether subjective-SES moderates the interaction between MCII and cigarette dependence, we regressed smoking reduction on dummy-coded intervention condition (0 = control, 1 = MCII), cigarette dependence (standardized), and subjective-SES (standardized), and their three-way interaction. We used a stepwise regression with multiple models for 1) each predictor, 2) their two-way interactions, and 3) the three-way interaction.

Regression coefficients for all steps of the model are reported in Table S2. In step 3, we found that the effect of condition depended on cigarette dependence and subjective-SES, as evidenced by the three-way interaction. This pattern indicates that the greater effectiveness of MCII (vs. control) at higher levels of cigarette dependence is further increased at higher levels of SES.

To better understand what this interaction means for high-dependence smokers, because we are interested in an intervention that works best with high task-difficulty, we followed up with linear contrasts. For each of the two contrasts, we ran the same regression model but with cigarette dependence centered one standard deviation above the mean, and SES centered either one standard deviation below (contrast 1) or above (contrast 2) the mean (see Figure S1). Per the first contrast, we found no evidence for an effect of MCII (vs. control) at a high level of dependence and a low level of SES, *b* = -.10, *SE* = .87, *t*(318) = -.11, *p* = .91, 95% CI [-.61, 2.37]. However, per the second contrast, we found that MCII (vs. control) increased smoking reduction at a high level of dependence and a high level of SES, *b* = 3.11 , *SE* = .95, *t*(318) = 3.29, *p* = .001, 95% CI [.41, 3.66]. In this exploratory analysis of data from a brief online self-regulation intervention for cigarette smoking behavior change, we found preliminary evidence that the effect of MCII on smoking reduction is moderated by SES. Specifically, MCII increased smoking reduction relative to the control strategy among highly cigarette dependent participants who were also high in subjective-SES, but not if they were low-SES. Although MCII shows promise for highly dependent smokers, it is unclear whether MCII is an effective online intervention for low-SES smokers. We conducted the present study to further test these results in a sample of moderate to heavy smokers. Unlike Mutter et al. (2020), we did not expect cigarette dependence to moderate the effects of MCII, because we pre-screened participants to exclude light smokers and therefore anticipated less variance in cigarette dependence.

## Association between SES and Stress

To better interpret our findings, we computed the correlation between subjective-SES and perceived stress in the Mutter et al. (2020) dataset, after applying the present study’s inclusion criterion of smoking at least 15 CPD at baseline. These variables were not significantly associated, *r*(100) = -.09, *p* = .35.

# Exploratory Analyses

## Time Spent by Condition

As a check, we compared the length of time participants in both conditions spent on the surveys, the time spent writing during the intervention, and the length of writing for the intervention. We report adjusted results for homogeneity of variance violations where applicable. MCII participants (*M* = 1390.43, *SD* = 701.34) took longer, in seconds, to complete the combined T0-T1 surveys than control participants (*M* = 799.04, *SD* = 365.80), *t*(246.57) = -9.51, *p* < .001, 95% CI [-713.29, -469.49], *d* = -1.06. During the intervention at T1, MCII participants (*M* = 277.85, *SD* = 184.62) spent more seconds writing than did control participants (*M* = 240.95, *SD* = 144.26), *t*(305.54) = -2.00, *p* = .05, 95% CI [-73.18, -0.62], *d* = -.22, and wrote using more characters (MCII: *M* = 1098.92, *SD* = 629.46; control: *M* = 527.75, *SD* = 386.74), *t*(271.55) = -9.88, *p* < .001, 95% CI [-685.03, -457.31], *d* = -1.09. Thus, although the control strategy was an active control condition with similar demands on participants to MCII, MCII was more time-consuming and pulled for more writing than the control strategy.

The time (in seconds) to complete the T2 survey, however, did not differ by condition (MCII: *M* = 153.10, *SD* = 79.38; control: *M* = 144.88, *SD* = 71.64), *t*(200) = -.77, *p* = .44, 95% CI [-29.21, 12.78], *d* = -.11.

## Adjusted Versions of Smoking Reduction Analyses

We did not find evidence for T1 between-condition differences in any mental health or demographic characteristic (*p*’s ≥ .19) (see Table S1). Because the MCII and control groups differed (*p* < .15) in baseline smoking start age, cigarette dependence, cue exposure/associative processes, and long-term expectations for reducing or quitting, we conducted versions of the primary smoking reduction analysis and the exploratory interaction analysis with perceived stress adjusting for these baseline variables (mean-centered) in an initial step. The adjustment variables explained a combined 2.7% of the variance in smoking reduction, *p* = .07. Smoking start age and long-term expectations were marginally significant predictors (*p*’s = .06), with participants who started smoking later in life reducing less, and participants with greater long-term expectations of success reducing more (cigarette dependence and cue exposure/associative processes: *p*’s ≥ .50).

Regarding the adjusted version of the primary smoking reduction analysis, condition and subjective-SES accounted for an additional .1% of the variance in smoking reduction (*p* = .90) in step 2, after adjusting for the baseline variables. We found no evidence for a main effect of condition or subjective-SES on cigarette reduction (condition: *b* = .22, *SE* = 0.48, *t*(318) = .47, *p* = .64, 95% CI [-.72, 1.17]; subjective-SES: *b* = .003, *SE* = .14, *t*(318) = .02, *p* = .98, 95% CI [-.27, .27]. In step 3, we found a marginally significant interaction effect of condition and subjective-SES on cigarette reduction, *b* = -.47, *SE* = .24, *t*(317) = -1.91, *p* = .06, 95% CI [-.95, .01], $\Delta R^{2}$ = .01. Adjusting for the baseline variables, the condition effect at low subjective-SES was marginally significant, with greater reduction in the MCII (vs. control) condition, *b* = 1.14, *SE* = .68, *t*(317) = 1.68, *p* = .09, 95% CI [-.19, 2.48]. The condition effect at high subjective-SES was in the opposite direction but nonsignificant, *b* = -.68, *SE* = .67, *t*(317) = -1.01, *p* = .31, 95% CI [-2.01, .64].

Regarding the adjusted version of the exploratory analysis involving perceived stress, condition and stress accounted for an additional .1% of the variance in smoking reduction (*p* = .86) in step 2, after adjusting for the baseline variables. We found no evidence for a main effect of condition or stress on cigarette reduction (condition: *b* = .23, *SE* = .48, *t*(318) = .48, *p* = .63, 95% CI [-.72, 1.18]; stress: *b* = .10, *SE* = .32, *t*(318) = .30, *p* = .76, 95% CI [-.53, .72]. In step 3, we found a significant interaction effect of condition and stress on cigarette reduction, *b* = 1.64, *SE* = .60, *t*(317) = 2.75, *p* = .006, 95% CI [.46, 2.81], $\Delta R^{2}$ = .02. Adjusting for the baseline variables, the condition effect at high stress was significant, with greater reduction in the MCII (vs. control) condition, *b* = 1.53, *SE* = .67, *t*(317) = 2.27, *p* = .02, 95% CI [.21, 2.85]. The condition effect at low stress was trending in the opposite direction, *b* = -1.11, *SE* = .68, *t*(317) = -1.63, *p* = .10, 95% CI [-2.46, .23].

## Robustness of Primary and Secondary Analyses

To assess the robustness of our primary and secondary analyses, we conducted two alternative versions: one in which we exclude participants who met the smoking inclusion criteria at pre-screening (T0) but smoked fewer than 15 cigarettes per day (CPD) at baseline (T1), and one in which we exclude influential observations.

### Primary and Secondary Analyses Sans T1 Light Smokers.

Although we pre-screened for moderate to heavy smokers at T0, we were curious if any eligible participants did not report smoking ≥ 15 CPD at T1. We filtered out any participants who did not report smoking at least 15 CPD at baseline and reran the primary and secondary analyses.

For our primary analysis excluding light smokers at T1, we found no main effect of condition on cigarette reduction, adjusting for subjective-SES: *b* = .08, *SE* = .61, *t* = .13, *p* = .90, 95% CI *_b_* [-1.12, 1.27]. We observed a trending main effect of subjective-SES on reduction, adjusting for condition: *b* = .36, *SE* = .26, *t* = 1.40, *p* = .16, 95% CI *_b_* [-.15, .86]. Additionally, we observed a trending interaction effect of condition and subjective-SES on reduction: *b* = -.62, *SE* = .35, t = -1.78, *p* = .08, 95% CI *_b_* [-1.31, .07]. We ran the planned contrasts at low (-1 SD) and high (+1 SD) levels of SES. We found a trending difference such that participants with low subjective-SES reduced smoking more in the MCII condition than the control condition: *b* = 1.29, *SE* = .81, *t* = 1.59, *p* = .11, 95% CI *_b_* [-.31, 2.89]. We did not find evidence at high levels of SES for a difference between MCII and the control: *b* = -1.14, *SE* = 1.01, *t* = -1.13, *p* = .26, 95% CI *_b_* [-3.12, .84].

Regarding our secondary analysis for smoking cessation, upon excluding the light smokers, we found no main effect of condition on smoking cessation, adjusting for subjective-SES: *b* = .67, *SE* = .88, Wald $\chi^{2}(1)$*=* .58, *p* = .45, 95% CI *_b_* [3.49, 11.00]. We observed a trending main effect of SES, adjusting for condition, *b* = .41, *SE* = .26, Wald $\chi^{2}(1)$*=* 2.54, *p* = .11, 95% CI *_b_* [.91, 2.50]. Lastly, we found no evidence for an interaction effect of condition and SES on cessation, *b* = .48, *SE* = .54, Wald $\chi^{2}(1)$*=* .78, *p* = .38, 95% CI *_b_* [.56, 4.69].

### Primary and Secondary Analyses Sans Influential Observations.

We used Cook’s distances as our indicator of influence. Our cutoff was .84, the critical value of the *F* distribution on (*k* + 1, *n* - *k* - 1) degrees of freedom with ɑ = .50, given three predictors (*k*) and a sample size (*n*) of 325 (Cohen et al., 2003).

For the primary smoking reduction analysis, no observations exceeded the cutoff value. However, for the secondary smoking cessation analysis, one observation exceeded the cutoff value. Without this observation, the main effects of condition and subjective-SES remained relatively unchanged, respectively: *b* = 1.56, *SE* = .81, Wald $\chi^{2}(1)$*=* 3.71, *p* = .05, 95% CI (e^b^ ) [.97, 23.25]; *b* = .48, *SE* = .18, Wald $\chi^{2}(1)$*=* 7.07, *p* < .01, 95% CI (e^b^ ) [1.13, 2.29]. Although it remained nonsignificant, the exclusion of this one influential point did cause the direction of the interaction effect to change: *b* = -.26, *SE* = .49, Wald $\chi^{2}(1)$ *=* .28, *p* = .60, 95% CI (e^b^ ) [.29, 2.03], Nagelkerke$\Delta R^{2}$ = .004.

## Steps Taken

In an exploratory analysis of whether MCII’s effect on taking an action step to reduce or quit was dependent on level of SES, we found no evidence for a main effect of condition, adjusting for subjective-SES, *b* = .02, *SE* = .24, Wald $\chi^{2}(1)$ *=* .01, *p* = .92, 95% CI (e^b^ ) [.64, 1.63]. We found a marginally significant main effect of subjective-SES on steps taken, adjusting for condition, such that higher-SES individuals were less likely to report having taken an action step, *b* = -.11, *SE* = .06, Wald $\chi^{2}(1)$ *=* 3.27, *p* = .07, 95% CI (e^b^ ) [.79, 1.01]. Together, condition and subjective-SES accounted for 1.4% of the variance in the likelihood of taking an action step, Nagelkerke$R^{2}$ = .014, $\chi^{2}(1)$*=* .004*, p* = .947.

In a related exploratory analysis, we created a sum score for each participant from the specific action steps, one through thirteen, and regressed this score on condition, SES, and their interaction. We found no evidence for a main effect of condition on the number of specific steps taken, adjusting for subjective-SES, *b* = .01, *SE* = .25, *t*(322) = .02, *p* = .98, 95% CI [-.48, .50]. We found no evidence for a main effect of subjective-SES on the number of steps taken, adjusting for condition, *b* = -.08, *SE* = .09, *t*(322) = -.93, *p* = .36, 95% CI [-.26, .09]. Additionally, we found no evidence for an interaction effect between condition and subjective-SES, *b* = .02, *SE* = .13, *t*(321) = .18, *p* = .86, 95% CI [-.23, .27]. For a list of the 13 steps and whether there was a condition-by-SES interaction effect in predicting the likelihood of taking each individual step, refer Table S3.

## Interaction with Cigarette Dependence

Mutter et al. (2020) observed that cigarette dependence (CDS) moderated the effect of condition on smoking reduction, with MCII improving reduction compared to the control strategy only among the highly dependent. In the present research, eligible participants smoked at least 15 cigarettes per day and therefore tended to be higher and more similar to one another, descriptively, in CDS (range [12, 24], *M* = 19.37, *SD* = 2.77) than those in Mutter et al. (2020; range [6, 25], *M* = 16.69, *SD* = 4.41). Although the stricter eligibility requirements truncated variability in CDS, we nonetheless examined whether dependence still moderated the condition effect on smoking reduction. In the present study, we found no evidence that CDS moderated the effect of condition (Table S4, Two-Way Interaction). Because our study was based on evidence for a three-way interaction with condition, CDS, and SES obtained using original data from Mutter et al. (2020), we also conducted a comparable analysis with the current data for comparison. We found no evidence for such a three-way interaction (Table S4, Three-Way Interaction). Only the two-way interaction between condition and SES approached significance, consistent with our primary analysis.

## Strategy Recall and Use

At T2, there was no difference in remembering learning a strategy between MCII participants (94 out of 100 remembered) and control participants (96 out of 101) remembered, χ^2^(1, *N* = 201) = .11, *p* = .74. There was also no difference in frequency of strategy use between those in the MCII condition (*M* = 3.34, *SD* = 1.88) and those in the control condition (*M* = 3.17, *SD* = 1.81), *t*(188) = -.65, *p* = .51, 95% CI [-.70, .36], *d* = -.09. Finally, participants indicated condition-specific recall of the intervention strategy, with those in the control condition favoring the correct “Reasons for Quitting/Reducing” option (*n* = 74 vs. *n*_MCII_ = 18), those in the MCII condition favoring the correct “WOOP” option (*n* = 59 vs. *n*_control_ = 3), neither group favoring the decoy “Text 2 Quit” option (*n*_MCII_ = 3, *n*_control_ = 1), and similar numbers selecting the “I don’t remember” option (*n*_MCII_ = 19, *n*_control_ = 23), χ^2^(3, *N* = 200) = 86.04, *p* < .001.

# Multiple Imputation

## General Approach

As an alternative to the no-change intent-to-treat approach for missing values, we also conducted a multiple imputation procedure for replacing missing scores with a series of predicted values based on a participant’s available scores on several “key” variables from the main analytic model and several “auxiliary” variables. This replacement procedure is done iteratively, with multiple simulated values to replace each missing value, so that variations in the imputed values reflect uncertainty in the simulation procedure and natural variability in scores (Schafer and Graham, 2002).

Multiple imputation assumes that data are missing at random (MAR), meaning that missingness depends on a person’s characteristics (i.e., missingness is not completely at random), but these relevant characteristics have been observed and can be accounted for (Cohen et al., 2003). However, the MAR assumption is difficult to test, and violations may have relatively little impact on results (Schafer and Graham, 2002). Nonetheless, our data contain several variables that predict missingness on the primary outcome variable of smoking reduction, indicating that at least some sources or correlates of missingness can be accounted for (see Table S5).

As a first step, we identified the key and auxiliary variables that served as predictors of missing values in the imputation procedure. Because our primary analysis used condition, mean-centered subjective-SES, and their interaction as predictors of smoking reduction, we considered these three predictors as our key variables. Additionally, we selected auxiliary variables according to these criteria: the variable must a) be measured prior to the intervention, b) be associated with smoking reduction, c) predict missingness in smoking reduction, and d) have no more than 5% missing scores (Collin et al., 2019). These criteria yielded seven auxiliary variables: frequency of tobacco use other than cigarettes, frequency of nicotine replacement use, short-term expectations of reducing or quitting smoking, symptoms of anxiety and depression (PHQ), racial/ethnic group identification, highest level of education, and average number of cigarettes smoked per day at T1 (for details, see next section).

With these key and auxiliary variables as predictors, missing scores for smoking reduction (missing: *n* = 135, 41.5%), smoking cessation (missing: *n* = 123, 37.8%), and whether any steps were taken to reduce or quit (missing: *n* = 124, 38.2%) were imputed 50 times per score, using linear or logistic regression as appropriate. This number of imputations (*m* = 50) is considered more than sufficient to remove noise from the estimates in many applications (Schafer and Graham, 2002). Additional details on our specifications in SPSS can be found in a later section of this document. Once the data were imputed, we re-ran our primary and secondary planned analyses, as well as the exploratory interaction with perceived stress.

## Variables Considered and Selected as Auxiliary Variables

Below is a complete list of variables considered as auxiliary variables for the multiple imputation procedure. Those selected as auxiliary variables, per the specified criteria, are in bold. The scale of measurement for each variable is listed in parentheses. (Note that treating the seven-point Likert-type scales as interval scales (“scale” type in SPSS) assumes that the scale points are equally spaced.) A superscript “W” next to a variable indicates that it has been Winsorized.

- Cigarette-smoking frequency (nominal, 2 groups)
- **Other forms of tobacco use frequency (ordinal)**
- **Nicotine replacement use frequency (ordinal)**
- Openness to reducing versus quitting smoking at T0 (nominal, 2 groups)
- **Short-term expectations of reducing or quitting (interval)**
- Short-term incentive value for reducing or quitting (interval)
- Long-term expectations of reducing or quitting (interval)
- Long-term incentive value for reducing or quitting (interval)
- Smoking-induced deprivation (nominal, 2 groups)
- Social/environmental goads subscale of the WISDM (interval)
- Cue exposure/associative processes subscale of the WISDM (interval)
- Nicotine dependence syndrome scale^W^ (ratio)
- Cigarette dependence scale (interval)
- Smoking start age^W^ (ratio)
- Quit attempt in past year (nominal, 2 groups)
- Length of (longest) past-year quit attempt, in days^W^ (ratio)
- Purchase method for cigarettes (nominal, 2 groups)
- Typical amount spent on one cigarette, in U.S. dollars^W^ (ratio)
- Goal of reducing versus quitting smoking at T1 (nominal, 2 groups)
- COVID-19-related perceived stress (interval)
- Perceived stress (interval)
- **Patient health questionnaire (PHQ) for symptoms of anxiety and depression (interval)**
- Gender (nominal, 3 groups)
- Age, in years^W^ (ratio)
- **Race/ethnicity (nominal, 9 groups reduced to 3 groups^^[[1]](#footnote-1)^*^)**
- Employment (nominal, 10 categories)
- **Highest level of education (ordinal)**
- Annual income (ordinal)
- Working from home due to COVID-19 (nominal, 2 groups)
- Uncertain employment status due to COVID-19 (nominal, 2 groups)
- **Average cigarettes per day (CPD) smoked at T1 (ratio)**

Table S5 displays how each of these auxiliary variables met the criteria for selection. All auxiliary variables were measured prior to the intervention, at either T0 or T1. To test for associations with smoking reduction scores, we computed Pearson’s correlations for interval or ratio variables, point-biserial correlations for dichotomous nominal variables, one-way ANOVAs with reduction as the outcome variable for nominal variables with more than two groups, and Kendall’s tau-*b* associations for ordinal variables (Khamis, 2008). With the exception of long-term expectations for reducing or quitting smoking, all variables that were *not* ultimately selected as auxiliary variables were removed from consideration for failing to reach even marginally significant associations with smoking reduction (i.e., all *p*s ≥ .10).

To test whether the remaining variables under consideration predict missingness in smoking reduction, we first computed a variable to capture missingness on smoking reduction (0 = *not missing/valid*, 1 = *missing*). Then, we computed point-biserial correlations for interval or ratio variables, chi-square tests of association for nominal variables, and Kendall’s tau-*b* associations for ordinal variables^^[[2]](#footnote-2)^*^. Long-term expectations was the only variable that failed to meet this criterion (*p* = .42). The remaining seven variables also met the final criterion, having no more than 5% missing scores (see Table S5).

## Multiple Imputation Specifications

In addition to those described in the General Approach section, we applied the following specifications in SPSS. Smoking reduction, smoking cessation, and steps taken were constrained to a dependent role in the imputation (i.e., they were not used to predict missing values on other variables). Intervention condition was constrained to an independent role (i.e., it was used as a predictor only, and not to be imputed; there were no missing condition values). The remaining variables involved were allowed to play both roles. Additionally, variables measured on an interval or ratio scale with set minimum and/or maximum values were constrained to impute values within that range. For example, short-term expectations was originally measured on a 1-to-7 Likert-type scale, so imputed values were to range from 1 to 7 (inclusive), and be rounded to the nearest integer. And, average CPD at T1 was set to have a minimum of 0, as smoking fewer than 0 cigarettes is not possible, but no maximum. The imputation sequence was as follows: condition, condition-by-SES interaction, subjective-SES, racial/ethnic group identification, frequency of tobacco use other than cigarettes, frequency of nicotine replacement use, highest level of education, short-term expectations, PHQ, average CPD at T1, smoking cessation, steps taken, then smoking reduction.

## Results for Main Analyses with Imputed Data

For the following analyses, estimates were computed for each of the 50 sets of imputed data, then pooled together. We report those pooled estimates here. Certain statistics, such as *R^2^*, are not available in SPSS for these pooled estimates and are therefore not reported. We first summarize the discrepancies in statistical significance between the multiple imputation (MI) approach and the last-observation-carried-forward (LOCF) approach from the main text, then report the full MI-based findings for the primary analysis, secondary analysis, and exploratory analysis involving stress.

For the primary analysis predicting smoking reduction, the condition contrast at low subjective-SES was trending (*p* < .15) per LOCF but marginally significant (*p* < .10) per MI. For the secondary analysis predicting smoking cessation, the main effect of condition was trending per LOCF but nonsignificant (nor trending) per MI. For the exploratory stress analysis, the interaction effect was significant (*p* < .05) per LOCF but marginally significant per MI, and the condition contrast at high stress was significant per LOCF but — despite an estimate similar in magnitude — nonsignificant (*p* = .17) per MI.

**Primary Analysis.** We tested whether subjective-SES moderated the effect of condition on smoking reduction using linear regression with the imputed data. We found no main effect of condition on cigarette reduction, adjusting for subjective-SES, *b* = .43, *SE* = .86, *t*(322) = .50, *p* = .62, 95% CI [-1.26, 2.13]. We found no evidence for a main effect of subjective-SES on cigarette reduction, adjusting for condition, *b* = .27, *SE* = .22, *t*(322) = 1.24, *p* = .22, 95% CI [-.16, .71]. We found a marginally significant interaction effect of condition and subjective-SES on cigarette reduction, such that on average MCII reduces smoking more than the control condition at low, but not high, levels of subjective-SES, *b* = -.77, *SE* = .46, *t*(321) = -1.70, *p* = .09, 95% CI [-1.67, .12].

Via linear contrasts, we observed a marginally significant difference such that participants with low subjective-SES (centered at the same level of subjective-SES as for the main version of this analysis; i.e., at 1.96 units below the mean) reduced smoking more in the MCII condition than the control condition, *b* = 1.94, *SE* = .99, *t*(320) = 1.97, *p* = .050, 95% CI [.002, 3.89]. We found no evidence for a difference in smoking reduction between MCII and the control intervention at high levels of subjective-SES, *b* = -1.09, *SE* = 1.43, *t*(320) = -.76, *p* = .45, 95% CI [-3.92, 1.75].

**Secondary Analysis.** We found no evidence for a main effect of condition on smoking cessation, adjusting for subjective-SES, *b* = .58, *SE* = .54, *p* = .29, 95% CI (e^b^ ) [.61, 5.19]. We observed a main effect of subjective-SES, adjusting for condition, such that high subjective-SES participants were more likely to report smoking cessation, *b* = .39, *SE* = .14, *p* = .004, 95% CI (e^b^ ) [1.13, 1.94]. Additionally, we found no evidence for an interaction effect of condition and subjective-SES on smoking cessation, *b* = .03, *SE* = .26, *p* = .91, 95% CI (e^b^ ) [.61, 1.74].

**Interaction with Perceived Stress.** Perceived stress was a marginally significant moderator of the condition effect on smoking reduction (*b* = 1.48, *SE* = .88, *t*(321) = 1.68, *p* = .09, 95% CI [-.25, 3.21]). Although the pattern was consistent with a greater effect of MCII among the highly stressed, the effect of condition on smoking reduction was not significant at either low (centered at the same level of stress as for the main version of this analysis; i.e., at .806 units below the mean) or high levels of stress (low: *b* = -.84, *SE* = 1.09, *t*(321) = -.77, *p* = .44, 95% CI [-2.98, 1.31]; high: *b* = 1.55, *SE* = 1.14, *t*(321) = 1.37, *p* = .17, 95% CI [-.69, 3.78]).

# Supplementary Tables

**Table S1.** Mental health and demographic characteristics, by condition. All these measures were taken at Time 1, prior to the intervention.

| **Characteristic** | **Control** | **MCII** |
| --- | --- | --- |
| Mean perceived stress (*SD*) | 2.06 (.77) | 2.05 (.84) |
| Mean COVID-related stress (*SD*) | 2.38 (.88) | 2.34 (.86) |
| Mean anxiety and depression (*SD*) | 5.32 (3.63) | 5.25 (3.64) |
| Mean age in years (*SD*) | 40.34 (11.50) | 40.61 (10.87) |
| Male, % (*n*) | 47.50 (76) | 45.73 (75) |
| Race/ethnicity, % (*n*) |  |  |
| European American/White/Caucasian/European descent | 75.63 (121) | 76.83 (126) |
| African American/Black/African descent | 6.88 (11) | 9.76 (16) |
| Hispanic American/Hispanic/Latino(a)(x) | 3.13 (5) | 3.66 (6) |
| American Indian/Native American/Alaskan Native | 6.88 (11) | 4.27 (7) |
| Asian American/Asian descent/Pacific Islander | 1.88 (3) | 1.83 (3) |
| Arab American/Middle Eastern descent/North African descent | .63 (1) | .00 (0) |
| Multiple racial/ethnic groups | 5.00 (8) | 3.05 (5) |
| Other | .00 (0) | .61 (1) |
| Mean subjective-SES (*SD*) | 5.23 (1.96) | 4.95 (1.96) |
| Mean annual income (*SD*) | 5.35 (1.82) | 5.48 (1.68) |
| Mean highest education level (*SD*) | 5.21 (1.74) | 5.00 (1.56) |
| Employment status, % (*n*) |  |  |
| Working full-time | 55.00 (88) | 56.10 (92) |
| Working part-time | 10.00 (16) | 8.54 (14) |
| Freelance/Self-employed | 8.13 (13) | 9.14 (15) |
| Homemaker | 1.88 (3) | 4.88 (8) |
| Retired | 3.75 (6) | 1.83 (3) |
| Disabled | 2.50 (4) | 5.00 (8) |
| Unemployed, seeking work | 7.50 (12) | 5.49 (9) |
| Unemployed, not seeking work | 2.50 (4) | 2.44 (4) |
| Student, full-time | 2.50 (4) | 1.83 (3) |
| Multiple employment groups | 6.25 (10) | 5.00 (8) |
| Working from home, % (*n*) | 57.50 (92) | 55.49 (91) |
| Uncertain employment during COVID, % (*n*) | 19.38 (31) | 21.95 (36) |

**Table S2.** Multiple regression predicting smoking reduction, in Mutter et al. (2020) dataset. CDS = cigarette dependence scale.

|  | **Predictor** | ***b* (*SE*)** | ***t*** | ***p*** | **95% CI *_b_*** |
| --- | --- | --- | --- | --- | --- |
| **Step 1**  *F*(3, 322) = 6.48, *p* < .001, *R^2^* = .06 | Condition | .52 (.46) | 1.14 | 0.26 | [-.38, 1.41] |
|  | CDS | .95 (.23) | 4.18 | < 0.001 | [.50, 1.40] |
|  | SES | .07 (.23) | .31 | 0.76 | [-.38, .52] |
| **Step 2**  Δ*F*(3, 319) = 1.84, *p* = .14, Δ*R^2^* = .02 | Condition | .51 (.46) | 1.12 | 0.27 | [-.39, 1.40] |
|  | CDS | .59 (.29) | 2.02 | 0.04 | [.02, 1.17] |
|  | SES | -.15 (.31) | -.48 | 0.63 | [-.76, .46] |
|  | Condition by CDS | .95 (.47) | 2.03 | 0.04 | [.03, 1.86] |
|  | Condition by SES | .58 (.46) | 1.25 | 0.21 | [-.33, 1.49] |
|  | CDS by SES | .09 (.23) | .39 | 0.70 | [-.36, .53] |
| **Step 3**  Δ*F*(1, 318) = 5.20, *p* = .02, Δ*R^2^* = .02 | Condition | .64 (.46) | 1.41 | 0.16 | [-.26, 1.54] |
|  | CDS | .59 (.29) | 2.02 | 0.04 | [.02, 1.16] |
|  | SES | -.22 (.31) | -.70 | 0.49 | [-.83, .40] |
|  | Condition by CDS | .86 (.47) | 1.86 | 0.06 | [-.05, 1.78] |
|  | Condition by SES | .57 (.46) | 1.23 | 0.22 | [-.34, 1.47] |
|  | CDS by SES | -.36 (.30) | -1.20 | 0.23 | [-.95, .23] |
|  | Condition by CDS by SES | 1.04 (.46) | 2.28 | 0.02 | [.14, 1.93] |

**Table S3.** Steps taken to reduce smoking. The condition-by-SES interaction effect results are from a binary logistic regression predicting having taken each step. Because there were 13 steps, we used a Bonferroni adjusted alpha of .0038 to determine statistical significance. None of the interaction effects met this criterion. Percentages are out of (*n* = 161) for the control group and (*n* = 164) for the MCII group.

|  |  | **Control** | **MCII** | **Condition-by-SES Interaction** | | |
| --- | --- | --- | --- | --- | --- | --- |
|  | **Step** | **% (*n*)** | **% (*n*)** | ***b* (*SE*)** | **Wald χ^2^(1)** | **95% CI(*e^b^*)** |
| 1 | Thought about and identified the triggers that cause me to smoke (e.g., feeling anxious, drinking alcohol, seeing someone else smoke) | 24.22 (39) | 22.56 (37) | -.02 (.14) | .03 | [.75, 1.28] |
| 2 | Used something like a coin or toothpick to keep my hands or mouth busy | 12.42 (20) | 11.59 (19) | -.20 (.18) | 1.13 | [.57, 1.18] |
| 3 | Done something to relieve stress or improve my mood besides smoking a cigarette (e.g., deep breathing, talking to a friend) | 13.66 (22) | 15.85 (26) | .00 (.16) | .00 | [.73, 1.37] |
| 4 | Intentionally kept myself busy with a task when a craving hit | 18.63 (30) | 22.56 (37) | -.03 (.15) | .03 | [.73, 1.30] |
| 5 | Treated myself to something pleasurable and/or relaxing instead of smoking | 5.59 (9) | 6.10 (10) | -.24 (.25) | .91 | [.49, 1.28] |
| 6 | Used a form of nicotine replacement therapy (e.g., patches, gum, lozenges) | 7.45 (12) | 8.54 (14) | -.18 (.22) | .68 | [.54, 1.28] |
| 7 | Tried to keep my energy level stable (e.g., by getting regular exercise, eating healthy snacks, or getting plenty of sleep at night) | 8.07 (13) | 10.37 (17) | -.13 (.21) | .43 | [.59, 1.31] |
| 8 | Thrown away smoking materials (e.g., cigarettes, lighters) | 5.59 (9) | 5.49 (9) | .59 (.26) | 5.03 | [1.08, 3.03] |
| 9 | Gotten rid of smoking reminders (e.g., by washing the smoke smell off of my clothing) | 5.59 (9) | 5.49 (9) | .11 (.25) | .20 | [.69, 1.82] |
| 10 | Used a quit smoking resource (e.g., an app, a website, a quit helpline) | 2.48 (4) | 3.05 (5) | .36 (.35) | 1.04 | [.719, 2.84] |
| 11 | Told friends and/or family that I'm reducing or quitting smoking | 8.70 (14) | 6.71 (11) | .24 (.22) | 1.22 | [.83, 1.94] |
| 12 | Used a medication (e.g., Chantix, Zyban) | 2.48 (4) | 3.05 (5) | .38 (.36) | 1.10 | [.72, 2.93] |
| 13 | Removed myself from situations where others are smoking | 9.32 (15) | 5.49 (9) | .18 (.23) | .66 | [.77, 1.87] |

**Table S4.** Regression estimates from exploratory analyses involving cigarette dependence as a predictor of smoking reduction. Estimates were derived from entering all predictors simultaneously in the model. CDS = cigarette dependence.

|  | ***b* (*SE*)** | ***t*** | ***p*** | **95% CI** |
| --- | --- | --- | --- | --- |
| **Two-Way Interaction Model**  *F*(3, 321) = .73, *p* = .53, *R^2^* = .007 |  |  |  |  |
| Condition | .21 (.48) | .44 | 0.66 | [-.74, 1.16] |
| CDS | .05 (.12) | .44 | 0.66 | [-.18, .29] |
| CDS x Condition | .12 (.18) | .66 | 0.51 | [-.23, .46] |
| **Three-Way Interaction Model**  *F*(7, 317) = .90, *p* = .51, *R^2^* = .019 |  |  |  |  |
| Condition | .14 (.51) | .28 | 0.78 | [-.86, 1.15] |
| CDS | .06 (.13) | .48 | 0.63 | [-.20, .32] |
| SES | .17 (.19) | .91 | 0.37 | [-.20, .54] |
| Condition x CDS | .03 (.19) | .14 | 0.89 | [-.35, .40] |
| Condition x SES | -.45 (.27) | -1.71 | 0.09 | [-.97, .07] |
| CDS x SES | .06 (.06) | .88 | 0.38 | [-.07, .18] |
| Condition x CDS x SES | -.03 (.09) | -.35 | 0.73 | [-.21, .15] |

**Table S5.** Characteristics of auxiliary variables for multiple imputation. Missingness refers to missingness on smoking reduction. T0 = pre-screening/Time 0. T1 = baseline/Time 1. *r*_τ_ = Kendall’s tau-*b*. *r* = Pearson’s correlation. *r_pb_* = point-biserial correlation. PHQ = Patient Health Questionnaire, which measures symptoms of anxiety and depression. CPD = cigarettes per day.

| **Variable** | **Measured at** | **Association with Reduction** | **Association with Missingness** | **Count (%) missing** |
| --- | --- | --- | --- | --- |
| Other tobacco use frequency | T0 | *r*_τ_(*N* = 190) = .10, *p* = 0.097 | *r*_τ_(*N* = 324) = .23, *p* < 0.001 | 1 (0.30) |
| Nicotine replacement frequency | T0 | *r*_τ_(*N* = 190) = .17, *p* = 0.006 | *r*_τ_(*N* = 324) = .22, *p* < 0.001 | 1 (0.30) |
| Short-term expectations | T1 | *r*(188) = .15, *p* = 0.04 | *r_pb_*(321) = .15, *p* = 0.006 | 2 (0.60) |
| PHQ | T1 | *r*(188) = .14, *p* = 0.06 | *r_pb_*(321) = .14, *p* = 0.01 | 2 (0.60) |
| Race/ethnicity (3-group) | T1 | original: *F*(7, 182) = 1.81, *MSE* = 26.61, *p* = 0.09  3-group: *F*(2, 187) = 5.48, *MSE* = 26.17, *p* = 0.005 | original: χ^2^(7) = 18.88, *p* = 0.009  3-group: χ^2^(2) = 12.30, *p* = 0.002 | 1 (0.30) |
| Education | T1 | *r*_τ_(*N* = 190) = .12, *p* = 0.03 | *r*_τ_(*N* = 324) = .23, *p* < 0.001 | 1 (0.30) |
| Average CPD | T1 | *r*(188) = .13, *p* = 0.07 | *r_pb_*(320) = -.23, *p* < 0.001 | 3 (0.90) |

# Supplementary Figures
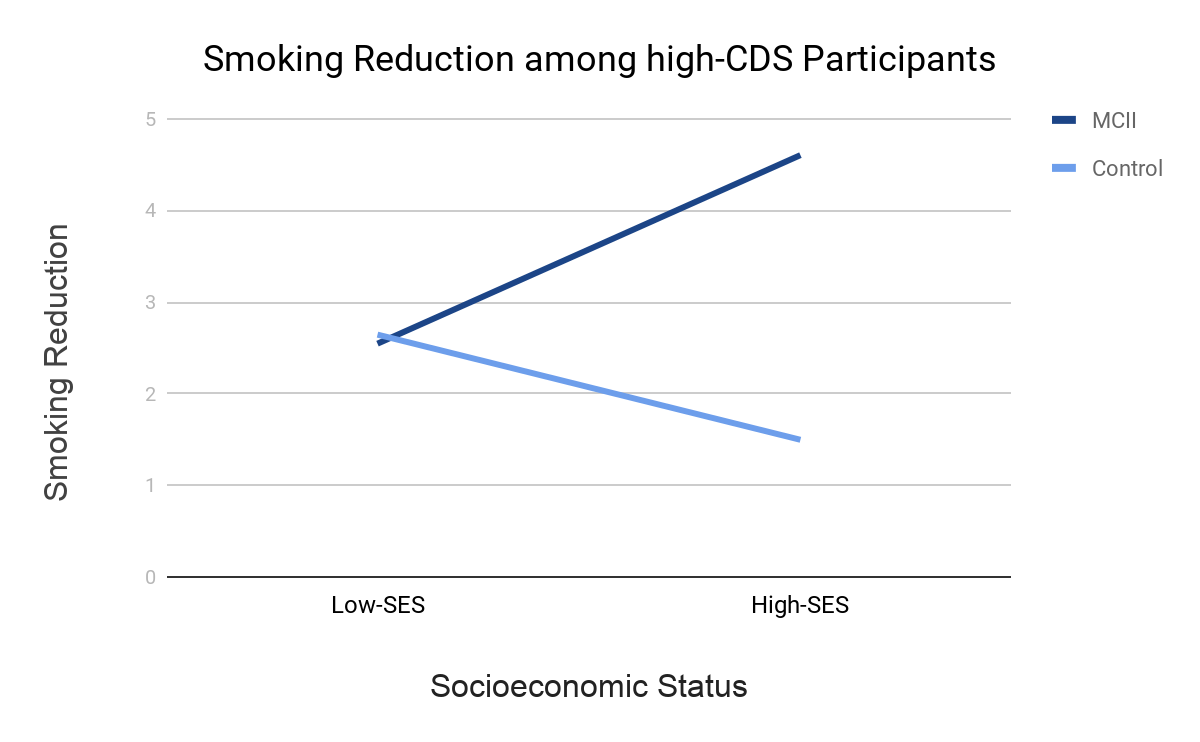

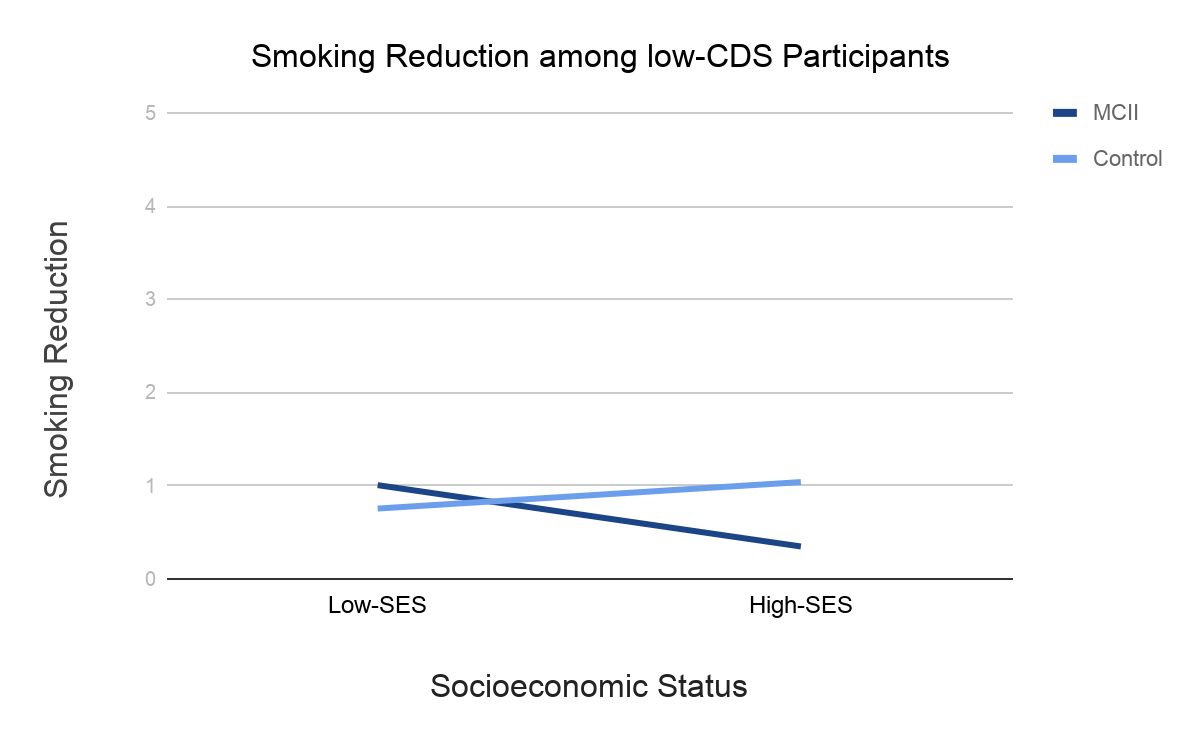


**Supplementary Figure 1.** Smoking reduction (cigarettes/day) in Mutter et al. (2020) dataset, by condition, subjective-SES, and cigarette dependence (CDS).

# References

Baggett, T. P., Lebrun-Harris, L. A., and Rigotti, N. A. (2013). "Homelessness, cigarette smoking and desire to quit: results from a US national study." *Addiction* 108 (11): 2009-18. <https://doi.org/10.1111/add.12292>.

Cohen, J., Cohen, P., West, S., and Aiken, L. (2003). "Outliers and multicollinearity: Diagnosing and solving regression problem II." *Applied multiple regression/correlation analysis for the behavioral sciences*: 390-430.

Collin, S. M., Norris, T., Joinson, C., Loades, M. E., Lewis, G., Stansfeld, S. A., and Crawley, E. (2019). "Depressive symptoms at age 9–13 and chronic disabling fatigue at age 16: A longitudinal study." *Journal of adolescence* 75: 123-129.

Etter, J. F., Le Houezec, J., and Perneger, T. V. (2003). "A self-administered questionnaire to measure dependence on cigarettes: the cigarette dependence scale." *Neuropsychopharmacology* 28 (2): 359-70. <https://doi.org/10.1038/sj.npp.1300030>.

Hoaglin, D. C., Mosteller, F., and Tukey, J. W. (2000). *Understanding robust and exploratory data analysis*. Vol. Sirsi) i9780471384915.

Khamis, H. (2008). "Measures of association: how to choose?" *Journal of Diagnostic Medical Sonography* 24 (3): 155-162.

Kroenke, K., Spitzer, R. L., Williams, J. B., and Löwe, B. (2009). "An ultra-brief screening scale for anxiety and depression: the PHQ-4." *Psychosomatics* 50 (6): 613-21. <https://doi.org/10.1176/appi.psy.50.6.613>.

Mutter, E. R., Oettingen, G., and Gollwitzer, P. M. (2020). "An online randomised controlled trial of mental contrasting with implementation intentions as a smoking behaviour change intervention." *Psychology & health* 35 (3): 318-345.

Piper, M. E., Piasecki, T. M., Federman, E. B., Bolt, D. M., Smith, S. S., Fiore, M. C., and Baker, T. B. (2004). "A multiple motives approach to tobacco dependence: the Wisconsin Inventory of Smoking Dependence Motives (WISDM-68)." *J Consult Clin Psychol* 72 (2): 139-54. <https://doi.org/10.1037/0022-006x.72.2.139>.

Rogers, E., Vargas, E., Rosen, M., Barrios-Barrios, M., Rana, M., Rezkalla, J., Rozon, R., Wysota, C., and Sherman, S. (2019). "Integrating Financial Coaching and Smoking Cessation Coaching to Reduce Health and Economic Disparities in Low-Income Smokers." APHA's 2019 Annual Meeting and Expo (Nov. 2-Nov. 6).

Schafer, J. L., and Graham, J. W. (2002). "Missing data: our view of the state of the art." *Psychological methods* 7 (2): 147.

Shiffman, S., Waters, A., and Hickcox, M. (2004). "The nicotine dependence syndrome scale: a multidimensional measure of nicotine dependence." *Nicotine Tob Res* 6 (2): 327-48. <https://doi.org/10.1080/1462220042000202481>.

Siahpush, M., Borland, R., and Yong, H. H. (2007). "Sociodemographic and psychosocial correlates of smoking-induced deprivation and its effect on quitting: findings from the International Tobacco Control Policy Evaluation Survey." *Tob Control* 16 (2): e2. <https://doi.org/10.1136/tc.2006.016279>.

1. ^*^ The original racial/ethnic group identification variable contained nine categories, eight of which were selected by at least one participant. When we conducted a two-way chi-square test of association between race/ethnicity and missingness on smoking reduction, seven of the cells had expected counts of less than five participants, reflecting the fact that some of the racial/ethnic groups had very low representation (e.g., only six participants identified as Asian American/Asian descent/Pacific Islander). To address this, we created a new version of the racial/ethnic group variable that retained the two most highly represented racial/ethnic groups (European American/White/Caucasian/European descent, *n* = 247, African American/Black/of African descent, *n* = 27) and collapsed the remaining groups into one “Other” category (*n* = 50). We then reevaluated this variable according to our criteria and found support for including it as an auxiliary variable (see Table S5). [↑](#footnote-ref-1)
2. ^*^ Per Khamis (2008), a rank-biserial correlation would be most appropriate to estimate the association between an ordinal variable (e.g., nicotine replacement use frequency) and a dichotomous variable (missingness). However, as SPSS does not have an option for computing a rank-biserial correlation, we instead computed Kendall’s tau-*b* as an approximation. [↑](#footnote-ref-2)
